# Measuring the iron content of dopaminergic neurons in substantia nigra with MRI relaxometry

**DOI:** 10.1016/j.neuroimage.2021.118255

**Published:** 2021-10-01

**Authors:** Malte Brammerloh, Markus Morawski, Isabel Friedrich, Tilo Reinert, Charlotte Lange, Primož Pelicon, Primož Vavpetič, Steffen Jankuhn, Carsten Jäger, Anneke Alkemade, Rawien Balesar, Kerrin Pine, Filippos Gavriilidis, Robert Trampel, Enrico Reimer, Thomas Arendt, Nikolaus Weiskopf, Evgeniya Kirilina

**Affiliations:** a Department of Neurophysics, Max Planck Institute for Human Cognitive and Brain Sciences, Stephanstr. 1a, Leipzig 04103, Germany; b International Max Planck Research School on Neuroscience of Communication: Function, Structure, and Plasticity; c Felix Bloch Institute for Solid State Physics, Faculty of Physics and Earth Sciences, Leipzig University, Linnéstr. 5, Leipzig 04103, Germany; d Paul Flechsig Institute of Brain Research, University of Leipzig, Liebigstr. 19, Leipzig, 04103, Germany; e Jožef Stefan Institute, Jamova 39, Ljubljana SI-1000, Slovenia; f Integrative Model-based Cognitive Neuroscience Research Unit, University of Amsterdam, Amsterdam, Nieuwe Achtergracht 129B, 1001 NK Amsterdam, The Netherlands; g The Netherlands Institute for Neuroscience, Institute of the Royal Netherlands Academy of Arts and Sciences, Amsterdam, Netherlands; h Center for Cognitive Neuroscience Berlin, Free University Berlin, Habelschwerdter Allee 45, Berlin, 14195, Germany

**Keywords:** Substantia nigra, Nigrosome, Quantitative MRI, Neuromelanin, Ion-beam microscopy, Iron-induced contrast

## Abstract

In Parkinson’s disease, the depletion of iron-rich dopaminergic neurons in nigrosome 1 of the *substantia nigra* precedes motor symptoms by two decades. Methods capable of monitoring this neuronal depletion, at an early disease stage, are needed for early diagnosis and treatment monitoring. Magnetic resonance imaging (MRI) is particularly suitable for this task due to its sensitivity to tissue microstructure and in particular, to iron. However, the exact mechanisms of MRI contrast in the *substantia nigra* are not well understood, hindering the development of powerful biomarkers. In the present report, we illuminate the contrast mechanisms in gradient and spin echo MR images in human nigrosome 1 by combining quantitative 3D iron histology and biophysical modeling with quantitative MRI on *post mortem* human brain tissue. We show that the dominant contribution to the effective transverse relaxation rate ($R_{2}^{*}$) in nigrosome 1 originates from iron accumulated in the neuromelanin of dopaminergic neurons. This contribution is appropriately described by a static dephasing approximation of the MRI signal. We demonstrate that the $R_{2}^{*}$ contribution from dopaminergic neurons reflects the product of cell density and cellular iron concentration. These results demonstrate that the *in vivo* monitoring of neuronal density and iron in nigrosome 1 may be feasible with MRI and provide directions for the development of biomarkers for an early detection of dopaminergic neuron depletion in Parkinson’s disease.

## Highlights

• Dopaminergic neurons dominate effective transverse relaxation in nigrosome 1.
• Ion beam microscopy reveals highest iron concentrations in dopaminergic neurons.
• Developed biophysical model links MRI parameters to cellular iron content.
• Ferritin- and neuromelanin-bound iron impact MRI parameters differently.
• Quantitative MRI provides a potential biomarker of iron in dopaminergic neurons.

## 1 Introduction

Several neurodegenerative diseases, including Parkinson’s disease (PD), involve pathologic iron accumulation, which is also potentially the cause (Friedrich, Reimann, Jankuhn, Kirilina, Stieler, Sonntag, Meijer, Weiskopf, Reinert, Arendt, Morawski, 2021, Ward, Zucca, Duyn, Crichton, Zecca, 2014). In PD, iron overload in *substantia nigra* dopaminergic neurons (DN) is followed by their depletion (Zucca et al., 2017), starting in neuron-rich nigrosome 1 (Damier, Hirsch, Agid, Graybiel, 1999, Damier, Hirsch, Agid, Graybiel, 1999). This neuronal depletion precedes motor symptoms of PD by nearly two decades. Yet, the majority of DN are irreversibly lost before PD diagnosis, which is based on motor symptoms (Agid, 1991; Kalia and Lang, 2015). *In vivo* methods capable of monitoring iron content and loss of DN would be highly desirable for early diagnosis and assessment of potential treatments.

Recent advances in magnetic resonance imaging (MRI) promise to provide such information, allowing a unique, noninvasive glimpse into cellular iron distribution (Duyn and Schenck, 2017; Edwards et al., 2018; Fukunaga et al., 2010; Sulzer et al., 2018). Importantly, several MRI parameters are known to differ between the *substantia nigra* (SN) of PD patients and healthy controls. Among them are the effective transverse relaxation time ($T_{2}^{*}$) and the intensity in $T_{2}^{*}$-weighted images ($T_{2}^{*}$-WI) (Kwon et al., 2012), local magnetic susceptibility (Langkammer et al., 2016), and the image intensity in an MRI sequence sensitive to neuromelanin, the main iron chelator in DN (Sasaki et al., 2006). In the SN of PD patients, the most striking change is the disappearance of the so-called swallow tail sign, an elongated structure with prolonged $T_{2}^{*}$ in between regions of lower $T_{2}^{*}$. The region of prolonged $T_{2}^{*}$ is often interpreted as nigrosome 1 (N1) (Blazejewska et al., 2013; Cheng et al., 2019; Lehéricy et al., 2014; Péran et al., 2010; Schwarz et al., 2014). In a population of patients with motor symptoms, the absence of this sign can be used to diagnose PD with a sensitivity of 100 % and a specificity of 95 % or higher (Cosottini et al., 2014; Schwarz et al., 2014). This high diagnostic power at a late disease stage has raised interest even further that MRI-based PD biomarkers may also be useful for early stage diagnostics.

Despite the wide-spread use of MRI for imaging SN, it is not well understood which tissue components actually contribute to the MRI contrast in SN. While multiple tissue components of SN induce transverse MRI relaxation (leading to contrast), iron is thought to be the primary contributor in the myelin-poor nigrosomes (Lee et al., 2018). Several studies have performed careful qualitative comparisons of MRI and histology on *post mortem* tissue from PD patients and healthy controls (Blazejewska et al., 2013; Lee, Baek, Chun, Lee, Cho, 2018, Lee, Baek, Kim, Huh, Lee, Cho, 2020; Sasaki et al., 2006), demonstrating that nigrosomes contrast with the surrounding SN tissue. Iron in the neuromelanin of DN, as well as in ferritin, the iron storage protein in brain tissue, was hypothesized to decisively impact relaxation (Lee, Baek, Chun, Lee, Cho, 2018, Lee, Baek, Kim, Huh, Lee, Cho, 2020; Zecca et al., 2004a). However, a quantitative link between MRI parameters in SN, SN’s cellular composition, and the cellular iron distribution is still missing. Quantitative information about the iron distribution in different cellular populations in SN is largely lacking (Morawski et al., 2005; Reinert, Fiedler, Morawski, Arendt, 2007, Reinert, Spemann, Morawski, Arendt, 2006). It is not clear whether neuromelanin or ferritin iron dominates the iron-induced MRI contrast in SN, particularly in the nigrosomes.

A strong quantitative link between MRI parameters and the cellular iron distribution is needed to enhance the specificity and interpretability of MRI as a biomarker. Despite providing descriptions of the effective transverse relaxation time of blood (Kiselev, Novikov, 2002, Kiselev, Novikov, 2018), the role of blood oxygen (Gagnon et al., 2015; Ulrich and Yablonskiy, 2016; Uludağ et al., 2009), and enabling blood vessel measurement (Troprés et al., 2001), current theory describing MR relaxation on a microscopic scale, induced by magnetic perturbers such as iron (Gagnon et al., 2015; Kiselev and Novikov, 2018; Yablonskiy and Haacke, 1994), is insufficient. It is necessary to apply such a theory to describe the relaxation resulting from iron-rich cells in the nigrosomes.

Herein, we address that need by building and validating a quantitative biophysical model of iron-induced MR relaxation in the nigrosomes of SN. The cellular iron distribution between DN and other tissue components in the nigrosomes was quantified by combining 3D quantitative iron histology, based on proton-induced X-ray emission microscopy (PIXE), and histochemistry on *post mortem* human tissue. The predominant contribution of iron to the transverse and effective transverse relaxation rates ($R_{2}=1/T_{2}$ and $R_{2}^{*}=1/T_{2}^{*}$) in the nigrosomes was quantified, using ultra-high resolution, quantitative MRI, and chemical iron extraction from tissue. Combining the obtained knowledge with biophysical modeling of the MRI signal, we demonstrate that iron accumulated in DN underlies the majority of iron-induced relaxation in N1 and provide an appropriate model for this contribution. Extrapolating the biophysical model, we show that assessing the iron content of DN *in vivo* is within reach of state-of-the-art MRI. We therefore provide the crucial quantitative link between MRI parameters and cellular iron distribution, which is an important step toward *in vivo* characterization of DN to enable PD diagnosis at an earlier stage of the disease.

## 2 Theoretical considerations

The iron in tissue contributes to the transverse and effective transverse relaxation rates through processes that can be categorized as molecular interactions on the nanoscale and dephasing due to a heterogeneous cellular iron distribution on the microscale (Eqs. (1), (2) Kiselev and Novikov (2018)). In order to interpret relaxation rates in SN and to link them to the cellular iron distribution, we estimated the impact of different relaxation processes from first principles and determined the most relevant ones. For readers interested in the details of our biophysical model, an analytical description of spin echo (SE) and gradient echo (GE) decays induced by nano- and microscale processes is presented in Section 3.1. The most important results for interpreting iron-induced MRI parameters and guiding the experiments are summarized here.

Notably, the relaxation processes on the nanometer and micrometer scale manifest themselves differently in $R_{2}^{*}$ and $R_{2}$. Molecular interactions with iron on the nanoscale induce very fast fluctuations of the water proton Larmor frequency, resulting in transverse relaxation. Such processes impact $R_{2}^{*}$ and $R_{2}$ equally, due to diffusion averaging effectively over the nanoscale distances between the iron-storage complexes. The nanoscale contributions to relaxation rates are determined by the concentration of iron stored in ferritin and neuromelanin averaged over the volume of interest (${\langle c_{\mathrm{Fe},\text{NM}}\rangle }_{V}$ and ${\langle c_{\mathrm{Fe},\text{FT}}\rangle }_{V}$, respectively; Eq. (3)) and are not dependent on the cellular iron distribution.

In contrast, the heterogeneous cellular distribution of iron on the microscale results in a perturbation of the Larmor frequency around iron-rich tissue components (such as iron-rich cells and fibers), which are not fully averaged out by water diffusion. Therefore, $R_{2}^{*}$ is impacted stronger than $R_{2}$, up to an exclusive contribution to $R_{2}^{*}$ in the static dephasing limit for large or well separated iron-rich structures (Eq. (8)). Hence, the microscale contribution is very sensitive to the cellular distribution of iron. In cases where the averaging due to water diffusion is negligible, the microscale relaxation rates can be determined from the Larmor frequency perturbation induced by iron (Eq. (8)). In the specific case of sparse iron-rich cells, $R_{2}^{*}$ is a highly informative biomarker: It is proportional to the susceptibility difference between the cells and their surrounding (Eq. (9) Yablonskiy and Haacke (1994)).

Importantly, iron stored in ferritin and neuromelanin contributes differently to relaxation rates both for nanoscale and microscale relaxation mechanisms, since these two iron binding forms differ with respect to their magnetic properties and accessibility to water (Brooks et al., 1998; Cho et al., 2004; Gossuin et al., 2000; Schäfer-Nolte, 2014; Trujillo et al., 2017; Zecca et al., 2004a).

To summarize, iron-induced $R_{2}^{*}$ and $R_{2}$ are driven by several mechanisms, dependent on different aspects of the cellular iron distribution. Estimating the dominating relaxation mechanism in the nigrosomes and quantifying the contribution of DN to $R_{2}^{*}$ and $R_{2}$ requires comprehensive knowledge of the quantitative 3D microscopic iron distribution in both chemical forms.

## 3 Materials and methods

### 3.1 Biophysical model of iron-induced transverse relaxation

Iron contributes to transverse and effective transverse relaxation rates ($R_{2}$ and $R_{2}^{*}$, respectively) through processes occurring at different temporal and spatial scales (Kiselev and Novikov, 2018). These processes can be categorized into molecular interactions on the nanoscale and dephasing due to a heterogeneous cellular iron distribution on the microscale (Kiselev and Novikov, 2018). This is a plausible assumption as the correlation times on the two scales differ by several orders of magnitude: Assuming a tissue diffusion coefficient of $D=1\mu m^{2}/\text{ms}$, the diffusion times $\tau_{D}=l^{2}/D$ across nano- (l=10nm) and microscale (l=10μm) distances are 100ns and 100ms, respectively. In case of statistical independence, the decays of both spin and gradient echo signals ($S_{\mathrm{GE}}$ and $S_{\mathrm{SE}}$) can be described as a product of decays induced by each process:

(1) $$\begin{array}{cc} S_{\mathrm{GE}}\left(T_{E}\right)= & \exp\left(-\int_{0}^{T_{E}}dt\;R_{2,\mathrm{nano}}^{*}\right)\cdot \exp\left(-\int_{0}^{T_{E}}dt\;R_{2,\mathrm{micro}}^{*}\right)\cdot \\ & \exp\left(-R_{2,\mathrm{other}}^{*}T_{E}\right), \end{array}$$

(2) $$\begin{array}{cc} S_{\mathrm{SE}}\left(T_{E}\right)= & \exp\left(-\int_{0}^{T_{E}}dt\;R_{2,\mathrm{nano}}\right)\cdot \exp\left(-\int_{0}^{T_{E}}dt\;R_{2,\mathrm{micro}}\right)\cdot \\ & \exp\left(-R_{2,\mathrm{other}}T_{E}\right), \end{array}$$

where $R_{2,\mathrm{nano}/\text{micro}}$ and $R_{2,\mathrm{nano}/\text{micro}}^{*}$ are the iron-induced transverse and effective transverse relaxation rates, respectively, resulting from processes on the nano- and microscale. They are in general time-dependent, allowing for non-exponential behavior. $R_{2,\mathrm{other}}$ and $R_{2,\mathrm{other}}^{*}$ are the relaxation rates induced by tissue components others than iron. Note that our model disregards the possibly complex relaxation processes on the intermediate length scale of several hundred nanometers. In Section 5.3, we discuss the plausibility of this assumption.

#### 3.1.1 Molecular interactions on the nanoscale

On the nanoscale, spin-spin interactions of water protons with iron electrons result in transverse MRI relaxation. Acting on the nanometer length scale, these processes depend on the iron binding site (iron spin state and water accessibility), but are independent of the cellular distribution of iron (Kiselev and Novikov, 2018). Since the diffusion time over the nanoscale distances is much smaller than the echo time of an MRI experiment, this relaxation mechanism results in a linear-exponential decay and contributes equally to transverse and effective transverse relaxation rates, i.e. $R_{2,\mathrm{nano}}=R_{2,\mathrm{nano}}^{*}$.

The contributions of ferritin- and neuromelanin-bound iron to the nanoscale transverse relaxation rate can be estimated from empirical relaxivities measured in ferritin and neuromelanin in vitro at room temperature, physiological pH, and a static magnetic field of 7T used in this study:

(3) $$R_{2,\mathrm{nano}}=R_{2,\mathrm{nano}}^{*}=r_{2,\mathrm{FT}}\cdot {\langle c_{\mathrm{Fe},\text{FT}}\rangle }_{V}+r_{2,\mathrm{NM}}\cdot {\langle c_{\mathrm{Fe},\text{NM}}\rangle }_{V}.$$

Here, $r_{2,\mathrm{FT}}=0.02\;s^{-1}/\left(\mu g/g\right)$ (Gossuin et al., 2000)^2^ and $r_{2,\mathrm{NM}}=0.8\;s^{-1}/\left(\mu g/g\right)$ (Trujillo et al., 2017)^3^are the relaxivities of iron in ferritin and neuromelanin, respectively, and ${\langle c_{\mathrm{Fe},\text{FT}}\rangle }_{V}$ and ${\langle c_{\mathrm{Fe},\text{NM}}\rangle }_{V}$ are the volume-averaged tissue iron concentrations of ferritin- and neuromelanin-bound iron, respectively, i.e. iron concentrations averaged over the volume of interest V.

#### 3.1.2 Heterogeneous cellular iron distribution on the microscale

The MRI signal from brain tissue is affected by dephasing due to magnetic tissue heterogeneity on the cellular microscale (Kiselev and Novikov, 2018; Yablonskiy and Haacke, 1994). In particular, the heterogeneous distribution of paramagnetic iron among different cell types (Morawski et al., 2015; Zecca et al., 2004b) strongly impacts the MRI signal. Larmor frequency perturbations caused by iron-rich cells induce MRI signal dephasing and therefore signal decay (Duyn and Schenck, 2017). Assuming a spatially varying concentrations of neuromelanin- and ferritin-bound iron $c_{\mathrm{Fe},\text{NM}}\left(r\right)$ and $c_{\mathrm{Fe},\text{FT}}\left(r\right)$, respectively, the resulting spatially varying magnetic volume susceptibility is

(4) $$\Delta \chi \left(r\right)=\rho \left(c_{\mathrm{Fe},\text{NM}}\left(r\right)\chi_{\mathrm{Fe},\text{NM}}+c_{\mathrm{Fe},\text{FT}}\left(r\right)\chi_{\mathrm{Fe},\text{FT}}\right).$$

Here, $\chi_{\mathrm{Fe},\text{NM}}$ and $\chi_{\mathrm{Fe},\text{FT}}$ are the effective mass susceptibilities of neuromelanin and ferritin-bound iron, respectively, and ρ the tissue density. This heterogeneous distribution of magnetic iron induces spatial variations of magnetic field and therefore of the proton Larmor frequency, according to Marques and Bowtell (2005)

(5) $$\frac{f(r)}{f_{0}}=\frac{1}{4\pi }\int \frac{\Delta \chi (r^{\prime })}{|r-r^{\prime }{|}^{3}}\left(3\frac{{\left(z-z^{\prime }\right)}^{2}}{|r-r^{\prime }{|}^{2}}-1\right)d^{3}r^{\prime },$$

where $f_{0}$ is the Larmor frequency, r=(x,y,z), and $r^{\prime }=\left(x^{\prime },y^{\prime },z^{\prime }\right)$.

The resulting relaxation rates depend on the spatial distribution of tissue iron and diffusion of water molecules through regions with a spatially varying Larmor frequency (Kiselev and Novikov, 2018). In the general case, the GE and SE decay contributions from microscale processes can be described by

(6) $$\begin{array}{ccc} & & \exp\left(-\int_{0}^{T_{E}}dt\;R_{2,\mathrm{micro}}^{*}\right)=\\ & & \;\;{\langle \exp\left(-2\pi i\int_{0}^{T_{E}}dt\;\Delta f\left(r\left(t\right)\right)\right)\rangle }_{r}, \end{array}$$

(7) $$\begin{array}{cc} \exp\left(-\int_{0}^{T_{E}}dt\;R_{2,\mathrm{micro}}\right)= & \\ 〈\exp(-2\pi i(\int_{0}^{T_{E}/2} & dt\;\Delta f\left(r\left(t\right)\right)-\int_{T_{E}/2}^{T_{E}}dt\;\Delta f\left(r\left(t\right)\right))){〉}_{r}, \end{array}$$

respectively, where Δf is the iron-induced Larmor frequency perturbation and r(t) the coordinate of a diffusing water proton spin. The averaging in Eqs. (6) and (7) is performed over the diffusion paths of all water protons within the MRI voxel, which cannot be performed analytically in the general case. Instead, numerical Monte Carlo simulations can predict MRI signal decays for arbitrary distributions of magnetic perturbers and tissue diffusion properties (Gagnon et al., 2015).

In the case of negligible diffusion, an analytical solution is available: the static dephasing approximation. Negligible diffusion means that the time scale of signal dephasing is much shorter than the diffusion time over the length scale of magnetic inhomogeneities (Yablonskiy and Haacke, 1994). In this case, the microscale contribution to the transverse relaxation rate $R_{2,\mathrm{micro}}$ is zero and only an effective transverse relaxation rate $R_{2,\mathrm{micro}}^{*}$ is induced. If the water protons remain static, the path integral in Eq. (6) simplifies to the Fourier transformation of the Larmor frequency probability density ρ(Δf) (Marques and Bowtell, 2005), which can be estimated from the intravoxel Larmor frequency histogram (Fig. 4A):

(8) $$\exp\left(-\int_{0}^{T_{E}}dt\;R_{2,\mathrm{micro}}^{*}\right)=\int_{-\infty }^{\infty }d\left(\Delta f\right)\;\rho \left(\Delta f\right)e^{-2\pi i\Delta ft}.$$

In the special case of Larmor frequency perturbations caused by localized magnetic inclusions of simple geometry (here, iron-rich dopaminergic neurons), the analytical solution of Eq. (8) provides a quantitative link between the susceptibility of DN and $R_{2,\mathrm{micro}}^{*}$. As was demonstrated by Yablonskiy and Haacke (1994), spherical magnetic inclusions contribute to $R_{2}^{*}$ according to

(9) $$R_{2,\mathrm{micro}}^{*}=\frac{2\pi }{9\sqrt{3}}\gamma B_{0}\cdot \zeta \Delta \chi ,$$

where ζ is the volume fraction of the magnetic inclusions and Δχ is the difference in susceptibility between the inclusions and the surrounding tissue. Eq. (9) is valid in the exponential decay regime for times greater than $\tau =1/2\omega_{L}\Delta \chi$, where $\omega_{L}$ is the Larmor frequency and Δχ the susceptibility difference between the spherical magnetic inclusion and its surrounding. Importantly, in this regime the contribution of magnetic inclusions to $R_{2}^{*}$ is proportional to the product of their volume fraction and their susceptibility difference to the surrounding tissue.

### 3.2 Software implementation

The biophysical model was predominantly implemented using the Python programming language (Python Software Foundation, https://www.python.org/). A previously published Monte Carlo simulation (Gagnon et al., 2015) was re-implemented in the C++ programming language and run with $10^{6}$ protons and a 0.1ms time step. We verified the convergence of the Monte Carlo simulation with these settings by comparing the predicted GE decay from three simulation runs with randomly generated proton starting positions. The standard deviation of the signals divided by their mean was less than 0.35% for all echo times used in GE imaging (up to 50ms, see Section 3.4. When comparing each of the runs with a simulation with ten times more protons, the latter differed less than 3 % to the former simulations. The diffusion constant was set to $D=1\mu m^{2}/ms$ *in vivo*. In *post mortem* tissue, D was set to $0.3\;\mu m^{2}/ms$. This was estimated from the mean diffusivity measured with diffusion weighted imaging on one sample. The simulations were informed with Larmor frequency shift maps with an isotropic resolution of 0.88micrometre (see Section 3.11). Relaxation rates were calculated with the same procedure as for experimental MRI data, using the experimental echo times for fitting (see Section 3.4).

### 3.3 *Post mortem* human brain tissue samples

Three midbrain samples (samples 1–3) including *substantia nigra* from human *post mortem* brains were provided by the Brain Banking Centre Leipzig of the German Brain Net (GZ 01GI9999-01GI0299), operated by Paul Flechsig Institute of Brain Research (Approval # 282-02). Sample 1 was donated by a 57-y-old male subject and contained bilateral SN. The sample was cut along the midline and the part containing the left SN used in the tissue iron extraction experiment, while the part containing the right SN was used for biophysical modeling. The samples 2 and 3 contained the left SN from a 86-y-old and a 61-y-old male subject, respectively. All tissue blocks contained the posterior-inferior half of SN. The causes of death of the donors of samples 1, 2, and 3 were liver failure, heart failure, and renal failure, respectively. Brain specimen were obtained at autopsy with prior informed consent and approved by the responsible authorities. The *post mortem* interval before fixation was less than 24h for all tissue samples. Following the standard Brain Bank procedures, blocks were immersion-fixed in 4% paraformaldehyde in phosphate buffered saline (PBS) at pH 7.4 for at least two weeks but less than three months to ensure complete fixation and avoid overly long detrimental fixation times. Prior to MRI experiments, tissue blocks were washed in PBS with 0.1% sodium azide for three days to remove formaldehyde residues from the tissue.

### 3.4 Quantitative MRI

Fixed tissue samples were placed in acrylic spheres of 6 cm diameter and immersed in Fomblin (Solvay Solexis, Bollate, Italy) to eliminate background MRI signal. MRI scanning was performed on a Siemens Magnetom 7T whole-body MRI scanner (Siemens Healthineers, Erlangen) using a custom-built two-channel quadrature coil designed for imaging small samples. 3D high resolution quantitative multi-parametric mapping (Weiskopf et al., 2013) was performed with the following parameters: A 3D multi-echo fast low-angle shot (FLASH) (Haase et al., 2011) with field of view (FOV) $32\times 32\times 25\;{\mathrm{mm}}^{3}$ for the first sample, $50\times 50\times 28\;{\mathrm{mm}}^{3}$ for the other samples; matrix size 144×144×112 for the first sample, 224×224×128 for the other samples (approximately 220m isotropic resolution for all samples); twelve echo times $T_{E}=4/7.34/10.68/\ldots /40.74\;\text{ms}$ recorded using bipolar readout; repetition time $T_{R}=60\;\text{ms}$; flip angle $\alpha =27^{\circ }$; bandwidth BW=344Hz/pixel. A single-slice 2D high resolution spin echo acquisition was performed with the following parameters: FOV $42\times 42\;{\mathrm{mm}}^{2}$ for the first sample, $28\times 28\;{\mathrm{mm}}^{2}$ for the other samples; slice thickness 0.6mm; matrix size 192×192 for the first sample, 128×128 for the other samples (219 micro meter isotropic in-plane resolution); six acquisitions with $T_{E}=15/25/35/45/55/75\;\text{ms}$ for the first sample, and with $T_{E}=11/16/25/37/56/83\;\mathrm{mm}$ for the other samples; $T_{R}=2\;s$; $\alpha =27^{\circ }$; BW=344 Hz/pixel. 3D ultra-high resolution $T_{2}^{*}$-WI was performed using a single-echo FLASH with the following parameters: FOV $46\times 37\times 14\;{\text{mm}}^{3}$; matrix size 896×728×287 (51×51×49μm${}^{3}$ resolution); $T_{E}=19.7\;\mathrm{mm}$; $T_{R}=180\;\mathrm{mm}$; $\alpha =48^{\circ }$; BW=40Hz/pixel; partial Fourier 6/8. All magnitude and phase images were reconstructed and stored. Quantitative parameter maps of $R_{2}^{*}$ and $R_{2}$ were calculated from the magnitude images using a linear-exponential fit with a Rician noise floor implemented in Python.

### 3.5 Iron extraction experiment

After the MRI acquisition, the posterior part of the left SN from sample 1 was soaked in a solution of 2 % deferoxamine and 2 % sodium dithionite for 15 days at $37{\;}^{\circ }C$ to remove iron from the tissue. The solution was changed every three days. No metals were present in the tissue after iron extraction, as confirmed by PIXE measurements. After iron extraction, the MRI acquisition was performed on this sample with the same parameters as before. Prior to MRI experiments, tissue blocks were washed in PBS with 0.1 % sodium azide for three days to remove deferoxamine and sodium dithionite from the tissue. The ROIs of N1 and N3 were segmented by an anatomy expert (M. M.) on the ultra-high resolution $T_{2}^{*}$-WI acquired before iron extraction (for a description of the nigrosome identification, see Section 3.7. A rigid landmark registration between the MRI data acquired before and after iron extraction was performed.

### 3.6 Histology and immunohistochemistry

**Fig. 1:**
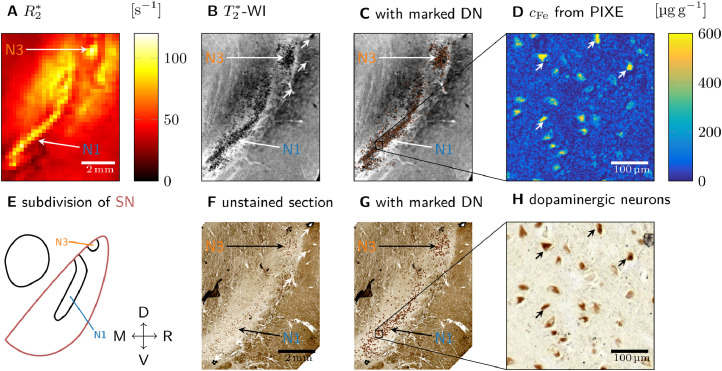
Quantitative histology and MRI (sample 1 shown, results for samples 2 and 3 are presented in Fig. S2). A: On a quantitative $R_{2}^{*}$ map of SN, nigrosomes N1 and N3 are visible as hyperintense areas. B: On ultra-high resolution $T_{2}^{*}$-WI of SN, granular hypointensities are visible in N1 and N3. C: DN marked on unstained sections (G) were overlaid on $T_{2}^{*}$-WI. Clusters of DN show a high spatial correspondence to regions with granular hypointensities. D: Quantitative iron map from a region in N1 obtained with PIXE. An increased iron concentration was observed in the neuromelanin domains within the cytoplasm of the DN. E: Subdivision of SN along medial (M), right (R), ventral (V), and dorsal (D) directions, showing an elongated N1 and a circular N3 (Damier et al., 1999b). F: An unstained tissue section including SN shows N1 and N3 as areas with increased density of neuromelanin-positive (brown) DN. The vascular landmarks used for co-registration of MRI and histology are marked with white arrows in B and in F. G: On the unstained tissue sections, each DN was marked with a brown dot for better visibility. H: Enlargement of the region of interest (ROI) within N1 marked in G, on which the PIXE measurement (D) was performed. Brown neuromelanin domains in DN were identified. Examples of identified DN are marked with arrows in D and in H.

Tissue blocks were embedded in paraffin (Histowax, SAV LP, Flintsbach) and cut into 10micrometer sections using a sliding microtome (Jung Histoslide 2000, Leica, Wetzlar). The cutting plane of the sections were tilted in the superior part to posterior by $20{}^{\circ }$, $45^{\circ }$, and $50^{\circ }$ for samples 1, 2, and 3, respectively. Block-face imaging was used for initial co-registration between histology and MRI. The sections were transferred to Superfrost®Plus glass slides (Thermo Fisher Scientific, Massachusetts). For sample 1, ten consecutive sections containing the right *substantia nigra* with visible neuromelanin-pigmented nigrosomes N1 and N3 (for a description of the nigrosome identification, see Section 3.7 were stained with Perls’ stain for iron in order to generate 3D quantitative iron maps. Deparaffinized sections were incubated for 2h at $37^{\circ }$C in Perls’ working solution, before they were washed in PBS and Tris–HCl. Prior to the 3,3’-diaminobenzidine (DAB) reaction, the sections were preincubated with 0.5mg DAB per ml Tris–HCl. After a 10 min DAB visualization reaction, the sections were washed in Tris–HCl, PBS, and distilled water before they were embedded in Entellan (Merck Millipore, Darmstadt). For further details on the staining process, see Friedrich et al. (2021). The sections were examined on an AxioScan.Z1 microscope (Zeiss, Jena) with a 20× objective lens (NA 0.5) with the same imaging parameters for all slides and no gamma correction. The images were precisely co-registered to the ultra-high resolution $T_{2}^{*}$-WI using the 3D Slicer software (https://www.slicer.org/). Using vessels as landmarks (Fig. 1B, F), a global similarity transformation between $T_{2}^{*}$-WI and unstained tissue sections was obtained, without further local refinement of the registration. For samples 2 and 3, a section was stained with Perls’ stain. For all samples, PIXE measurements were performed on the sections adjacent to the Perls’ stained sections. Consecutive sections were stained with Luxol fast blue to localize myelinated fibers, with anti-tyrosine hydroxylase (TH) antibody, and with anti-calbindin antibody for nigrosome verification.

### 3.7 Nigrosome delineation

Nigrosome 1 was segmented on histological sections following the widely used definition introduced by Damier et al. (1999b). First, the nigrosomes were identified as areas with low calbindin and high TH immunoreaction on all three samples (Figs. S1, S2). Next, the nigrosomes N1 and N3 were assigned based on their morphology and location following Damier et al. (1999b). Nigrosome N1 was identified as the largest nigrosome extending along the medioventral-dorsolateral axis (Figs. S1, S2G1-3). N3 was identified as a circular nigrosome at the dorsolateral end of SN of sample 1 (Fig. S2).

### 3.8 PIXE iron quantification

PIXE was used to acquire quantitative iron maps. Sections from all samples were deparaffinized, embedded in mounting medium (DePeX, Merck Millipore, Darmstadt), and subsequently placed into aluminum frames. Prior to PIXE, light microscopy was performed on the framed sections using an Axio Imager 2 microscope (Zeiss, Jena). The images were registered to ultra-high resolution $T_{2}^{*}$-WI as above. For sample 1, PIXE was performed at the Leipzig ion beam laboratory (LIPSION, Leipzig University, Leipzig) using a proton beam of 2.25 MeV and 0.5 nA with a diameter of 0.8micrometer. It locally knocked out inner shell electrons, leading to element-specific X-ray emission. Rutherford backscattering spectra were recorded for absolute concentration calculations. PIXE was performed on four ROIs in N1 with the following parameters: matrix size 1000×1000/1000×1000/500×1500/1600×400; FOV $800\times 800/400\times 400/400\times 1600/1600\times 400\;\mu m^{2}$; deposited charge 3.1/6.7/2.3/6.7μC. For samples 2 and 3, PIXE was performed at the Microanalytical Center (Department for Low and Medium Energy Physics, Jozef Stefan Institute, Ljubljana) using a proton beam of 3.03MeV and 100pA to 150pA with a diameter of 1.5m. The measurement parameters were: matrix size 256×256 for both; FOV $560\times 560/400\times 400\;\mu m^{2}$; deposited charge 10.23/6.45μC. Quantitative iron and sulfur maps were obtained using the GeoPIXE II software (CSIRO, Clayton), following Morawski et al. (2015). The locations of PIXE measurements within histological slices for all three samples are indicated on (Fig. S2D). These elemental maps were corrected to account for tissue shrinkage during paraffin embedding. A volume shrinkage factor of (0.76±0.02) was found by comparing the distance between vessels on ultra-high resolution $T_{2}^{*}$-WI on sample 1 with their distance in histology.

### 3.9 Iron quantification in neuromelanin

Light microscopy and PIXE were combined to determine the iron concentration in the neuromelanin of dopaminergic neurons and in ferritin outside of neuromelanin clusters. Neuromelanin clusters were identified on microscopy images as brown-pigmented domains, which most DN contain, especially the ones vulnerable in PD (Herrero et al., 1993). Neuromelanin clusters were segmented from the background, artifacts, and structures of no interest on microscopy images using a machine learning approach. We trained a random forest classifier using the WEKA trainable segmentation (Arganda-Carreras et al., 2017) implemented in FIJI. The resulting neuromelanin probability maps were binarized by thresholding at 50 % and subsequently performing a morphological opening with a $2\times 2\;\mu m^{2}$ kernel to remove small masking artifacts using the SimpleITK library. Microscopy images were co-registered to the PIXE measurements using elemental sulfur maps on which the sulfur-containing neuromelanin were visible (Fig. S4). To this end, the sulfur maps were binarized to obtain binary maps of neuromelanin clusters with high sulfur concentration. These sulfur-based neuromelanin masks were then co-registered to the neuromelanin masks obtained from light microscopy using an affine registration with ANTs (Avants et al., 2011).

The iron concentrations associated with neuromelanin (${\langle c_{\mathrm{Fe}}\rangle }_{A_{\mathrm{NM}}}$) and ferritin (${\langle c_{\mathrm{Fe}}\rangle }_{A_{\mathrm{FT}}}$) were estimated by averaging quantitative PIXE iron maps over the area inside ($A_{\mathrm{NM}}$) and outside ($A_{\mathrm{FT}}=A\setminus A_{\mathrm{NM}}$) of the neuromelanin masks, respectively. We assumed that the iron outside the neuromelanin mask is predominantly in ferritin while the iron inside the neuromelanin mask is mainly bound to neuromelanin, neglecting iron in other chemical forms. The overlap of the PIXE measurement areas on sample 1 (Fig. S1D1) was taken into account in the analysis by first averaging over the overlapping areas and second over the whole measurement area.

### 3.10 Generation of 3D quantitative iron maps within a voxel

3D quantitative iron maps of N1 were obtained by calibrating semi-quantitative iron maps generated from Perls’ stain with iron concentrations from PIXE, and subsequent co-registration. Semi-quantitative iron maps were obtained from microscopy images of sections stained with Perls’ for iron by applying the Lambert Beer law to the blue color channel, which showed the highest dynamic range. Next, quantitative maps of the iron concentration associated with neuromelanin in DN and ferritin outside of DN were generated by a separate calibration of semi-quantitative iron maps: The iron concentration in DN was set to the value extracted from quantitative PIXE iron maps using the subset of DN located directly adjacent to the semi-quantitative iron map’s volume. Outside of DN, the mean of the semi-quantitative iron maps in the region of the PIXE measurement areas in N1 in sample 1 (Fig. S1D1) was set to the iron concentration in ferritin from PIXE. A 3D quantitative iron map of N1 was obtained by co-registration of quantitative iron maps in an ROI containing a part of N1, encompassing a volume of $2.5\times 2.3\times 0.1\;{\mathrm{mm}}^{3}$. To this end, a rigid registration with shared DN on adjacent sections as landmarks was performed. The volume was cropped to a DN-rich area spanning over four voxels of high resolution quantitative MRI parameter maps in N1, i.e.$440\times 440\;\mu m^{2}$.

### 3.11 Informing the biophysical model

A susceptibility map was calculated from the 3D quantitative iron map using Eq. (4), by separately scaling iron concentrations in neuromelanin and ferritin with the effective susceptibilities of neuromelanin-bound iron ((3.3ppb/(g/g), see Supplementary Information) and ferritin-bound iron (1.3ppb/(g/g) Schenck (1992), respectively. For converting volume to mass susceptibility, we used a tissue density of $1\;{g/\text{cm}}^{3}$. The susceptibility map was transformed to an evenly spaced coordinate grid with a resolution of 0.88micrometer using third-order B-spline interpolation.

The 3D Larmor frequency shift in N1, used in Monte Carlo simulations (Fig. 5C, D) as well as to determine the Larmor frequency histogram (Fig. 5A), was obtained by convolving the 3D quantitative susceptibility map with a dipole kernel, using Eq. (5) in Fourier space, which simplifies the calculation (Marques and Bowtell, 2005). The 3D Larmor frequency shift in N1 was calculated at the same resolution as the susceptibility map, i.e. 0.88micrometre isotropic. For modeling the microscale relaxation induced by only iron in DN, the iron concentration outside of DN was set to the average concentration of ferritin-bound iron.

### 3.12 Generation of 2D quantitative iron maps within a section

Quantitative concentration maps of iron bound to neuromelanin were generated using optical density in neuromelanin clusters on unstained sections, calibrated with PIXE iron concentration measurements for samples 2 and 3. To obtain optical density maps, first gray-scale images were calculated by averaging the color values in RGB microscopy images of unstained sections. Next, optical density (OD) maps were obtained from the gray-scale image (GS) *via*

(10) $$OD=-\log(GS/GS_{\mathrm{bg}}),$$

where $GS_{\mathrm{bg}}$ was the gray-scale image value averaged over a region without neuromelanin pigmentation. To confine the optical density maps to neuromelanin clusters, the obtained OD maps were subsequently multiplied with the neuromelanin masks (see Section 3.9. The relation between OD and iron concentration in neuromelanin obtained from PIXE was examined by correlating these values averaged over individual DN in the PIXE measurement areas (Fig. S8). Quantitative maps of neuromelanin-bound iron covering entire histological section (Fig. 6C, G) were generated by transforming OD using data from a linear fit to the OD and PIXE neuromelanin-bound iron concentration of samples 2 and 3 (Fig. S8). In the linear fit, the PIXE neuromelanin-bound iron concentration was obtained by subtracting the average iron concentration found outside of the DN from the value inside of DN.

## 4 Results

### 4.1 Enhanced $R_{2}^{*}$ in the nigrosomes is induced by iron

In this section, we show that iron is the main contributor to effective transverse relaxation in the nigrosomes by (i) a qualitative comparison between MRI contrasts in *post mortem* SN tissue and histology and (ii) a quantitative analysis of the iron-induced contribution to $R_{2}^{*}$ and $R_{2}$ in a tissue iron extraction experiment.

**Fig. 2:**
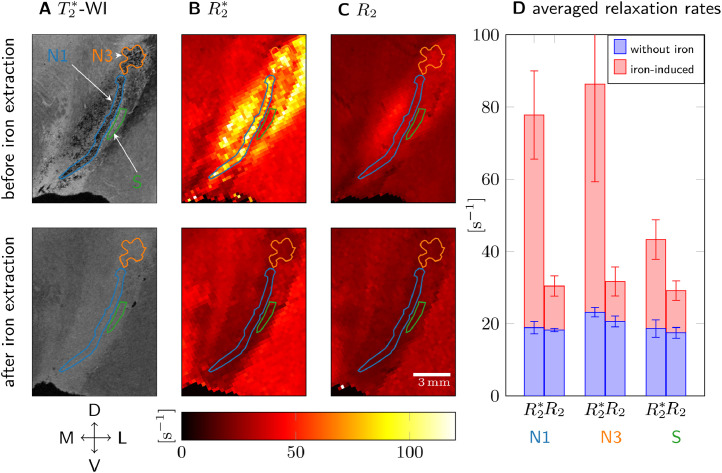
Transverse and effective transverse relaxation before (top row) and after chemical iron extraction (bottom) from sample 1. A: Granular hypointensities, used to delineate N1 and N3, disappeared after iron removal on 50 m resolution $T_{2}^{*}$-WI. B: On quantitative $R_{2}^{*}$ maps, the contrast between N1, N3, and the surrounding tissue (ROI S) was lost after iron extraction. C: On quantitative $R_{2}$ maps, no contrast between N1, N3, and S was observed before and after iron extraction. D: $R_{2}^{*}$ and $R_{2}$ averaged over ROIs N1, N3, and S before iron extraction (red plus blue bar) and after (blue bar) are shown. The difference in relaxation rates before and after iron extraction (red bar) is hence the iron-induced relaxation rate. Iron induced five times more $R_{2}^{*}$ than $R_{2}$ in N1 and N3, in S two times more $R_{2}^{*}$ than $R_{2}$. After iron extraction, $R_{2}^{*}$ and $R_{2}$ were almost equal in N1, N3, and S. The error bars indicate the standard deviation in the ROI. Anatomical directions are indicated as in Fig. 1, exchanging right (R) with left (L) while keeping the direction of the medial-lateral axis for easier comparison between the Figures.

To examine the origin of effective transverse relaxation in the nigrosomes qualitatively, we compared quantitative MRI acquired at 7T to histology and quantitative iron mapping on three tissue blocks containing SN (sample 1: Fig. 1, Fig. 2; samples 2 and 3: Fig. S1). High resolution $R_{2}^{*}$ and $R_{2}$ maps, ultra-high resolution $T_{2}^{*}$-WI, and histology were precisely registered using vascular landmarks (marked with asterisks for sample 1 in Fig. 1B, C). The histologically defined N1 area appeared as a hyperintense stripe on quantitative $R_{2}^{*}$ maps of all three tissue samples, showing high contrast to the surrounding SN tissue (Figs. 1A, 2 B, S2B1-3). The histologically defined N3 appeared similarly as a hyperintense circular area on quantitative $R_{2}^{*}$ maps of sample 1 (Figs. 1A). On ultra-high resolution $T_{2}^{*}$-WI of all three samples, granular hypointensities were visible in N1, suggesting the presence of magnetic field perturbers with size smaller than and separation greater than the length of the voxel edge in the $T_{2}^{*}$-WI acquisition of approximataley 50 micro meter (e.g., Fig. 1B). Areas of granular hypointensities on $T_{2}^{*}$-WI showed high overlap with nigrosomes defined using histological definition (Figs. 1C, S3). Quantitative iron maps obtained with PIXE on each of the three samples revealed microscopic spots of increased iron concentration in the nigral areas of enhanced $R_{2}^{*}$ (Figs. 1E; S2A, C). These hot spots were identified as neuromelanin-rich domains within DN in all samples (Figs. 1F; S2B, D). Combining this finding with the MRI results, we hypothesized that DN containing iron-rich neuromelanin are the microscopic magnetic perturbers causing increased $R_{2}^{*}$ in the nigrosomes.

To corroborate the above interpretation and quantify the iron-induced $R_{2}^{*}$ and $R_{2}$ in the nigrosomes, we analyzed quantitative MRI data acquired before and after chemical iron extraction of the tissue of one side of sample 1 (Fig. 2, Table 1). For this specimen, the contralateral side of the sample described in Fig. 1, N1 and N3 were delineated based on the distribution of granular hypointensities, which strongly resembled the distribution observed in Fig. 1. Before iron extraction, strong $R_{2}^{*}$ contrast was observed between the nigrosomes and the surrounding tissue (S), with significantly higher $R_{2}^{*}$ values in the nigrosomes (Fig. 2B, D). Nevertheless, no contrast between the nigrosomes and the surrounding tissue was observed in $R_{2}$ maps (Fig. 2C, D). $R_{2}$ values were much smaller than $R_{2}^{*}$ values.

**Table 1:** Relaxation rates $R_{2}$ and $R_{2}^{*}$ before and after tissue iron extraction averaged over ROIs in nigrosomes N1 and N3 and surrounding tissue S for sample 1 (see Fig. 2A for ROI definitions). The error is given as the standard deviation in the ROI.

ROI	before iron extraction		after iron extraction		iron-induced		
	$R_{2}^{*}$ [$s^{-1}$]	$R_{2}$ [$s^{-1}$]	$R_{2}^{*}$ [$s^{-1}$]	$R_{2}$ [$s^{-1}$]	$R_{2}^{*}$ [$s^{-1}$]	$R_{2}$ [$s^{-1}$]	$R_{2}^{*}-R_{2}$ [$s^{-1}$]
N1	77.8±12.1	30.4±2.4	18.9±1.7	18.2±1.5	58.9±12.2	12.2±2.8	46.7±12.5
N3	86.3±27.0	31.7±3.8	23.2±1.3	20.6±1.4	63.1±27.0	11.1±4.0	52.0±27.3
S	43.3±5.0	29.1±2.3	18.7±2.5	17.4±1.5	24.6±5.6	11.7±2.8	12.9±6.3

Iron extraction strongly reduced the $R_{2}^{*}$ values in the nigrosomes (Table 1). After extraction, neither the contrast between the nigrosomes and the surrounding tissue (Fig. 2B, D) nor the granular hypointensities in the nigrosomes on $T_{2}^{*}$-WI were visible (Fig. 2A). $R_{2}$ relaxation rates were somewhat reduced in N1, N3, and S after iron extraction (Table 1, Fig. 2C, D). Averaged $R_{2}^{*}$ and $R_{2}$ were similar in the nigrosomes after tissue iron extraction (Fig. 2D). In N1 and N3, the iron-induced contribution to $R_{2}^{*}$, estimated as a difference in relaxation rates before and after iron extraction, was almost five times higher than the iron-induced $R_{2}$ contribution. This observation points towards static dephasing as the dominant iron-induced relaxation mechanism.

### 4.2 Iron concentration is highest in the small DN somata but overall there is more ferritin-bound iron on the exterior

In this section, we quantify the 3D microscopic iron distribution in nigrosome N1 using a combination of classical histology and PIXE. The 3D microscopic iron maps were used to (i) determine the distribution of iron between dopaminergic neurons and other tissue components in N1 and (ii) to inform our biophysical model of iron-induced MRI contrast (see Section 4.3.

**Fig. 3:**
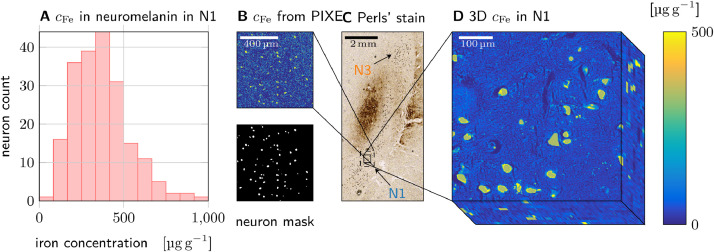
Quantitative iron histology of N1 in sample 1. A: Histogram of local iron concentrations found in neuromelanin domains in N1. B: Quantitative iron concentration maps obtained with PIXE on an unstained section (top) were masked using neuromelanin maps (bottom) to obtain the local concentration of iron bound to ferritin and neuromelanin (other PIXE measurement ares indicated in Fig. S2D1). C: N1 is visible as a stripe of high DN density on a section stained with Perls’ solution for iron. D: A 3D quantitative iron map of N1 was generated by calibrating and co-registering 10 adjacent sections stained with Perls’ solution for iron. This volume was used for biophysical model-ing (see Section 4.3).

Quantitative cellular iron concentration maps were obtained using PIXE (sample 1: Fig. 3; samples 2 and 3: Figs. S5, S6). The concentration of iron bound in two chemical forms was determined from these maps, assuming that iron within neuromelanin masks ($A_{\mathrm{NM}}$) is mainly bound in neuromelanin and outside of neuromelanin ($A_{\mathrm{FT}}$) mainly in ferritin (Table 2). Histograms of iron concentrations in neuromelanin averaged over individual neuromelanin clusters of DN were generated by using masks of the neuromelanin in the DN’s somata (sample 1: Fig. 3A, other samples: Fig. S6; Fig. 3B).

**Table 2:** Local iron concentration associated with neuromelanin (NM) and ferritin (FT) averaged over PIXE measurement areas in different samples. The concentration error is given as the standard error of mean (SEM) in the masked region in the PIXE iron maps. The error of the NM volume fraction $\zeta_{\mathrm{NM}}$ is given as SEM over PIXE measurement areas for the first sample, on which PIXE was performed on several ROIs.

sample	${\langle c_{\mathrm{Fe}}\rangle }_{A_{\mathrm{NM}}}$ [μg g${}^{-1}$]	${\langle c_{\mathrm{Fe}}\rangle }_{A_{\mathrm{FT}}}$ [μg g${}^{-1}$]	$\zeta_{\mathrm{NM}}$	NM iron fraction	$R_{2,\mathrm{micro},\text{DN}}^{*}$ (Eq. (9)) [s${}^{-1}$]
1	387±5	56±3	1.97±0.06 %	12.2±0.6 %	23.5 ± 0.4
2	671 ± 11	184 ± 0.8	3.5 %	11.8 ± 0.2 %	51.7 ± 1
3	1356 ± 11	451 ± 3	13.5 %	32.0 ± 0.3 %	390.0 ± 3.4

In all samples, a much greater local iron concentration was found in the neuromelanin within the DN’s somata, compared to ferritin-bound iron in the surrounding tissue (Table 2). However, as neuromelanin occupied only a fraction of the volume, only a minority of tissue iron was associated with neuromelanin (12 % to 32 %, Table 2). A strong variation of the local iron concentration in DN was found between neurons in each sample as well as between samples (Fig. S3).

For sample 1, a quantitative 3D microscopic iron concentration map of N1 was generated (Fig. 3D). The 3D map, spanning over several MRI voxels within N1, was obtained from co-registration of ten adjacent sections stained with Perls’ solution for iron and calibration with PIXE data. This 3D map of N1 was made quantitative by calibrating the underlying Perls’ stains with the iron concentration in neuromelanin and ferritin from PIXE data on sample 1 (Table 2, Fig. 3B, C, D).

The tissue iron concentration in neuromelanin and ferritin averaged over the volume of interest, necessary for predicting nanoscale relaxation rates, was estimated from the 3D iron concentration map. In neuromelanin and ferritin, they were ${\langle c_{\mathrm{Fe},\text{NM}}\rangle }_{V}=\zeta_{\mathrm{NM}}\cdot {\langle c_{\mathrm{Fe}}\rangle }_{V_{\mathrm{NM}}}=8.9\pm 0.2\;\mu {g\;g}^{-1}$ and ${\langle c_{\mathrm{Fe},\text{FT}}\rangle }_{V}=\left(1-\zeta_{\mathrm{NM}}\right)\cdot {\langle c_{\mathrm{Fe}}\rangle }_{V_{\mathrm{FT}}}=\left(51.1\pm 3.0\right)\;\mu g\;g^{-1}$, respectively, where $V_{\mathrm{NM}}$ and $V_{\mathrm{FT}}$ are the volumes inside and outside of the neuromelanin masks. These volume-averaged tissue iron concentrations differ slightly from the values reported for the PIXE measurements (Table 2), because the averages were taken over different ROIs (Figs. 3C, S2D1).

### 4.3 Microscopic iron distribution causes the majority of the iron-induced $R_{2}^{*}$ in N1, which is accurately described by a static dephasing approximation

As we have determined all necessary parameters for the biophysical model, we proceed with estimating the iron-induced relaxation rates originating from nanoscale and microscale processes. We identified the dominant contribution and appropriate theoretical description by comparing theoretical predictions with experimental data obtained before and after iron extraction from the tissue.

#### 4.3.1 Molecular interactions on the nanoscale

The nanoscale contributions of neuromelanin- and ferritin-bound iron were estimated to be $R_{2,\mathrm{nano},\text{NM}}=\left(7.54\pm 0.11\right)\;s^{-1}$ and $R_{2,\mathrm{nano},\text{FT}}=\left(1.14\pm 0.07\right)\;s^{-1}$, respectively, using Eq. (3). Interestingly, despite the fact that most iron is bound in ferritin, neuromelanin-bound iron in DN contributes dominantly to nanoscale relaxation due to its higher relaxivity. The total predicted nanoscale contribution $R_{2,\mathrm{nano}}^{*}=R_{2,\mathrm{nano}}=\left(8.67\pm 0.13\right)\;s^{-1}$ is much lower than the iron-induced $R_{2}^{*}\left(\left(42\pm 11\right)\;s^{-1}\right)$, but comparable to the iron-induced $R_{2}$ (($11.3\pm 1.8)\;s^{-1})$ in this volume. The experimental values were calculated as the difference between the measured relaxation rates in the MRI voxels corresponding to the location of 3D quantitative iron map and the non-iron-induced relaxation rates averaged over N1 from the iron extraction experiment, which was performed on the contralateral side of the same sample. Hence, the nanoscale relaxation is not the dominant relaxation mechanism for $R_{2}^{*}$, but may explain the observed iron-induced $R_{2}$ result.

#### 4.3.2 Heterogeneous cellular iron distribution on the microscale

**Fig. 4:**
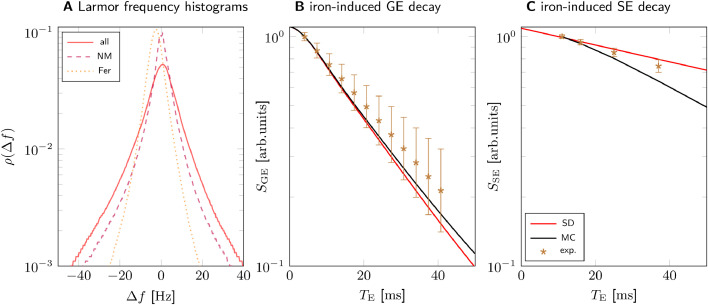
Modeling iron-induced microscale relaxation in N1 for sample 1. A: Larmor frequency shift histograms for all iron (solid), iron in neuromelanin (NM, dashed) and iron in ferritin (FT, dotted) show that iron in neuromelanin contributes most of the spectral width, which causes static dephasing (SD) decay. B: The GE signal decay predicted using SD is in good agreement with Monte Carlo simulations (MC) and experimental data. C: The SE signal decay predicted with MC somewhat overestimates the experimental SE decay. The predicted nanoscale relaxation rates were added to the shown iron-induced signal decays in B and C. The experimental data shown in B and C are experimentally derived iron-induced decays, calculated by subtracting the non-iron-induced $R_{2}$ and $R_{2}^{*}$ relaxation rates in N1 (Table 1) from the $R_{2}^{*}$ and $R_{2}$ rates averaged over the voxels corresponding to the 3D quantitative iron map, respectively. The experimentally derived and simulated iron-induced signal decays were normalized to their values at the first echo time ($T_{E}=4\;\mathrm{ms}$ for GE, $T_{E}=11\;\mathrm{ms}$ for SE) to compensate for possible non-exponential decay at shorter echo times. The error bars indicate the SEM of experimental relaxation rates.

Contributions of the microscopic heterogeneous cellular iron distribution to $R_{2}^{*}$ and $R_{2}$ were estimated from Monte Carlo simulations of water diffusion and the analytic static dephasing approximations of the MRI signal. In both approaches, the iron-induced Larmor frequency shift (Fig. S7) obtained from the 3D quantitative iron map (Fig. 3D) was used. A Monte Carlo simulation of water diffusion within this 3D Larmor frequency shift map predicted iron-induced GE and SE signal decays according to Eqs. (6) and (7). For the static dephasing approximation, the iron-induced $R_{2}^{*}$ was calculated from the histogram of the intravoxel iron-induced Larmor frequency perturbation (Fig. 4A). This histogram was numerically Fourier transformed to obtain the iron-induced GE signal decay (Eq. (8), Fig. 4B).

The predictions of the Monte Carlo simulations were in good agreement with the experimental data for $R_{2}^{*}$ and slightly overestimated $R_{2}$ (Fig. 4B, C). The error of the predicted relaxation rates was estimated from the residuals of the linear fit, as this was far larger than the error of the used volume-averaged tissue iron concentrations (Table 2). For the comparison, the predicted nanoscale relaxation rates were added to the microscale decay, while the non-iron-induced relaxation rate from the iron extraction experiments was subtracted from the experimental relaxation rate. The excellent agreement between the $R_{2}^{*}$ estimates indicates that our model captures iron-induced effective transverse relaxation accurately. The static dephasing approximation agreed very well with Monte Carlo simulations of the GE decay and the experimental $R_{2}^{*}$. From this good match, we can conclude that static dephasing contributes strongest to the effective transverse relaxation in N1.

### 4.4 DN are the main cellular source of iron-induced $R_{2}^{*}$ in N1

In this section, we use the developed biophysical model to estimate the contribution of dopaminergic neurons to effective transverse relaxation rates in nigrosome 1 in order to asses the sensitivity and specificity of $R_{2}^{*}$ to this cell type.

**Fig. 5:**
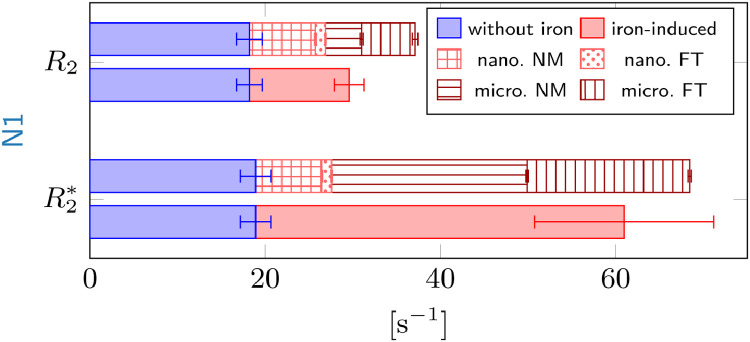
Comparison of predictions (patterned) to experimental $R_{2}$ and $R_{2}^{*}$ relaxation rates (solid color) in N1 of sample 1. The iron-induced relaxation rates (solid red) were obtained by subtracting the non-iron-induced relaxation rates in N1 from the iron extraction experiment (solid blue) from the relaxation rates measured in the volume corresponding to the 3D iron map. Top: The sum of the predicted nano- and microscale $R_{2}$ rates (patterned) in N1 somewhat overestimates the iron-induced $R_{2}$. Neuromelanin- (NM) and ferritin-bound (FT) iron contributes equally to the microscale $R_{2}$ relaxation rate (dark red), while neuromelanin dominates the nanoscale relaxation rates (light red). Bottom: In N1, the sum of the predictions of $R_{2}^{*}$ is in agreement with the experimental iron-induced $R_{2}^{*}$ within the SEM indicated by the error bar. The contribution of neuromelanin-bound iron to microscale $R_{2}^{*}$ (micro. NM, horizontal stripes) dominates. The contribution of ferritin-bound iron to microscale $R_{2}^{*}$ was estimated by subtracting the $R_{2}^{*}$ from neuromelanin-bound iron from the $R_{2}^{*}$ predicted for all iron. (For interpretation of the references to colour in this figure legend, the reader is referred to the web version of this article.)

The total $R_{2}^{*}$ and $R_{2}$ relaxation rates in N1 were estimated by adding the iron-induced relaxation rates from nano- and microscale mechanisms to the non-iron-induced relaxation rates averaged over N1 from the iron extraction experiment (Fig. 5). Predicted relaxation rates agreed well with experimental values: For $R_{2}^{*}$, the sum of the predicted iron-induced $R_{2}^{*}$ and measured non-iron-induced $R_{2}^{*}$ in N1, $\left(68.4\pm 1.8\right)\;s^{-1}$, was within the standard error of the mean of the experimental $R_{2}^{*}$ of $\left(61\pm 11\right)\;s^{-1}$. For $R_{2}$, the sum of the predicted iron-induced $R_{2}$ and measured non-iron-induced $R_{2}$ was $\left(37.1\pm 1.6\right)\;s^{-1}$, somewhat overestimating the experimentally determined $R_{2}$ of $\left(29.6\pm 0.9\right)\;s^{-1}$.

According to our simulations, $R_{2}^{*}$ is the parameter most sensitive to iron in DN somata. The microscale contribution from only the neuromelanin-bound iron in DN of $R_{2}^{*}=\left(22.32\pm 0.15\right)\;s^{-1}$ was predicted using a Monte Carlo simulation. This value agrees well with the analytic prediction of $R_{2}^{*}=\left(20.6\pm 0.4\right)\;s^{-1}$ for spherical iron-rich cells in static dephasing Eq. (9)). The linear regime of the static dephasing approximation applies, in which Eq. ((9) is valid, as the experimental echo times are larger than τ≈0.2mm, which was calculated using the iron concentration values from the 3D iron concentration map.

As static dephasing theory accurately models the GE decay, $R_{2}^{*}$ induced by iron in DN somata can be predicted analytically and is proportional to the susceptibility difference between DN and the surrounding tissue Eq. (9). In the case of iron-rich DN in N1, this susceptibility difference is approximately proportional to the concentration of neuromelanin-bound iron: Neuromelanin’s susceptibility per iron load is almost three times higher than that of ferritin, and the local iron concentration in neuromelanin is more than six times higher. Hence, the $R_{2}^{*}$ contribution from DN is a linear function of their volume-averaged tissue iron concentrations, i.e. the product of the average iron load of DN and the neuronal density $\zeta_{\mathrm{NM}}$. An effective microscale relaxivity $r_{2,\mathrm{NM}}^{*}$ can be assigned to iron in neuromelanin *via* Eq. (9):

(11) $$r_{2,\mathrm{NM}}^{*}\approx \frac{2\pi }{9\sqrt{3}}\gamma B_{0}\chi_{\mathrm{NM}}\approx 2.5\;s^{-1}\mu g^{-1}g.$$

Adding the nanoscale contribution of neuromelanin-bound iron, iron in DN caused (43.6±0.6)% of the total $R_{2}^{*}$ and (60.2±1.2)% of the iron-induced $R_{2}^{*}$. $R_{2}$ was less sensitive to iron in DN, which caused (31.4±1.8)% of the total $R_{2}$ and (61.7±1.53)% of the iron-induced $R_{2}$.

Interestingly, iron-induced $R_{2}^{*}$ and $R_{2}$ are two times more affected by neuromelanin-bound iron than the iron-induced bulk susceptibility: Iron in DN’s neuromelanin contributes merely (29.3±0.4)% to the iron-induced bulk susceptibility, as calculated by dividing the product of the DN’s volume fraction of 2.6% and their average susceptibility of (1111±15)ppb by the average susceptibility in the volume of the 3D quantitative iron map of (99±5)ppb.

### 4.5 Potential MR-based biomarkers of nigral integrity

In this section, we use the understanding of the impact of iron in DN on effective transverse and transverse relaxation rates in N1 to propose two potential MRI-based biomarkers of the volume-averaged iron concentration within dopaminergic neurons in nigrosome 1.

The first biomarker of the iron in the somata of dopaminergic neurons is derived from the difference between the effective transverse relaxation rate and the transverse relaxation rate in N1 ($R_{2}^{*}-R_{2}$). According to our results, this parameter is completely driven by iron and on the order of $50\;s^{-1}$ (Table 1, of which about 60 % are contributed by iron in DN somata *via* microscale relaxation (Fig. 5). The volume-averaged tissue iron concentration in DN may be approximated by

(12) $$c_{\mathrm{Fe},\text{NM},1}=\frac{\left(R_{2}^{*}-R_{2}\right)}{r_{2,\mathrm{NM}}^{*}}\approx 0.4\left(R_{2}^{*}-R_{2}\right)\;s\mu {gg}^{-1},$$

using the microscale relaxivity from Eq. (11). This relation holds if the microscale contribution of ferritin-bound iron to $R_{2}^{*}$ is approximately equal to the sum of the microscale contributions of neuromelanin- and ferritin-bound iron to $R_{2}$ (Fig. 5).

The second biomarker is the difference in $R_{2}^{*}$ between N1 and the directly surrounding tissue (e.g., area S in Fig. 2). This parameter is analytically linked to the volume-averaged tissue iron concentration in DN, if the contribution of ferritin-bound iron is approximately uniform in N1 and the surrounding tissue. In this case, the volume-averaged tissue iron concentration in DN can be estimated by

(13) $$\begin{array}{ccc} c_{\mathrm{Fe},\text{NM},2} & = & \frac{\left(R_{2}^{*}{|}_{N1}-R_{2}^{*}{|}_{S}\right)}{r_{2,\mathrm{NM}}+r_{2,\mathrm{NM}}^{*}}\\ & \approx & 0.3\cdot \left(R_{2}^{*}{|}_{N1}-R_{2}^{*}{|}_{S}\right)\;s\mu gg^{-1}. \end{array}$$

**Fig. 6:**
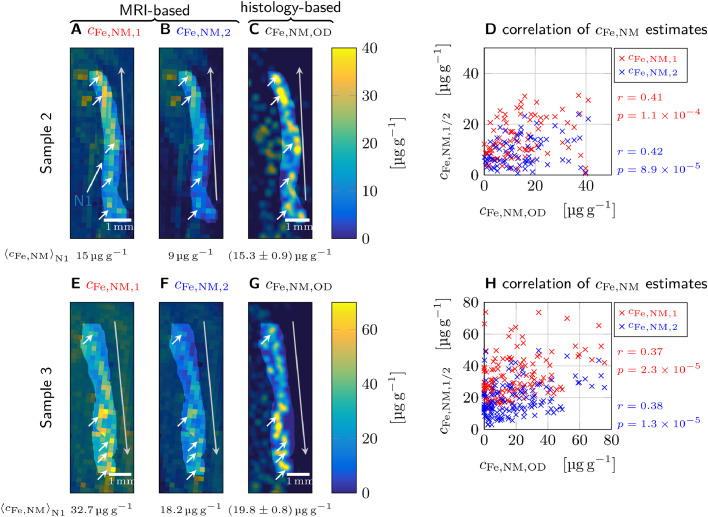
Validating relaxometry-based biomarkers of the volume-averaged tissue iron concentration in neuromelanin (${\langle c_{\mathrm{Fe}}\rangle }_{V}$) in N1 with histology for samples 2 (A-D) and 3 (E-H). A, E: The first MRI-based biomarker of neuromelanin-bound iron, $c_{\mathrm{Fe},\text{NM},1}$ (Eq. (12)) is derived from the difference between the $R_{2}^{*}$ and $R_{2}$ in N1. It shows several hot spots (white arrows) in N1 as well as a gradient of iron concentrations along the extension of N1 (gray arrow points toward increased concentrations). B, F: The second biomarker, $c_{\mathrm{Fe},\text{NM},2}$ (Eq. (13)), is based on the difference in $R_{2}^{*}$ between N1 and the directly surrounding tissue (e.g. area S in Fig. 2). It shows similar hot spots and gradients as in A and E, respectively, but overall lower iron concentrations. C, G: A map of $c_{\mathrm{Fe},\text{NM},\text{OD}}$ estimated from the optical density of neuromelanin clusters shows a similar spatial distribution of hot spots and gradients as in A, B and E, F, respectively, but is less homogeneous than the MRI-based maps. The averaged iron concentrations over the N1 area are given below the maps. The error is given as the standard error of mean, which was calculated from the errors of the linear fit between optical density and neuromelanin-bound iron concentration (Fig. S8), treating each area of the average DN size as an independent measurement. D, H: Significant correlations ($p<1.2\times 10^{-4}$) between the estimates of $c_{\mathrm{Fe},\text{NM},1}$, $c_{\mathrm{Fe},\text{NM},2}$, and $c_{\mathrm{Fe},\text{NM},\text{OD}}$ were found (Pearson correlation coefficients r and associated p-values are given next to the plots).

We validated both biomarkers of iron in DN in N1 on histological sections of sample 2 and 3, which are independent of data from sample 1 used for biophysical modeling Fig. 6. For sample 2 and 3, the volume-averaged tissue iron concentration in neuromelanin was estimated using Eq. (12) (Fig. 6A, E) and Eq. (13) (Fig. 6B, F) and quantitative $R_{2}^{*}$ and $R_{2}$ maps (Fig. S2 B2-3, C2-3). As an independent reference, the volume-averaged tissue iron concentration in neuromelanin was estimated from histology using a linear relation between PIXE iron concentrations and the optical density of neuromelanin clusters on unstained sections derived in the supplementary information (Fig. S8). For both samples 2 and 3, the MRI-based iron concentration maps showed a similar spatial distribution of iron-rich hot spots in N1 compared to the histological estimate, as well as a similar overall gradient. Both biomarkers’ predictions of the iron concentration in neuromelanin averaged over the N1 area were within 60 % of the value derived from the histological estimate (Fig. 6). The biomarkers show consistently a moderate, but highly significant ($p<1.2\times 10^{-4}$) correlation with the histological estimate of the volume-averaged tissue iron concentration in neuromelanin. The Pearson correlation coefficients of both biomarkers with the histological estimate were approximately the same.

### 4.6 Nigrosome integrity assessment is feasible with MRI in vivo

In this section, we examine theoretically how high the contribution of DN to $R_{2}^{*}$ in N1 would be in *in vivo* MRI. To this end, we extrapolated our finding from *post mortem* tissue to the *in vivo* MRI case by accounting for differences in temperature and tissue diffusion properties.

The body temperature *in vivo* as compared to room temperature in our *post mortem* experiments leads to a decreased iron-induced relaxation rate due to the reduced magnetic susceptibility of iron at higher temperature. Since the static dephasing contribution described by Eq. (8) scales linearly with magnetic susceptibility, and the susceptibility of iron is inversely proportional to the temperature, we expect a 5 % decrease of the iron-induced microscale $R_{2}^{*}$ *in vivo*.^4^

Additionally, the higher diffusivity *in vivo* shifts the microscale relaxation regime in the direction of motional narrowing. While this effect may decrease the relaxation contribution of iron, making $R_{2}^{*}$ less sensitive to this contribution, our model predicts that the microscale relaxation regime *in vivo* is still close to static dephasing (Fig. S5): a Monte Carlo simulation predicted $R_{2}^{*}=\left(37.7\pm 0.3\right)\;s^{-1}$, while the prediction of the static dephasing model was $R_{2}^{*}=\left(40.7\pm 0.06\right)\;s^{-1}$. The combined effect of decreased susceptibility and faster diffusion was 7.8 % less $R_{2}^{*}$ *in vivo*, which was estimated from Monte Carlo simulations (Fig. S5). Importantly, thus our model predicts that also *in vivo*, $R_{2}^{*}$ is a parameter sensitive to the volume averaged iron concentration in DN.

The nanoscale $R_{2}$ induced by ferritin-bound iron was reported to decrease by 15 % due to a temperature increase from room to body temperature (Gossuin et al., 2000). For neuromelanin-bound iron, no such data was published, but a similar decrease in nanoscale $R_{2}$ is expected.

While the increased temperature and diffusion constant *in vivo* decrease iron’s contribution to $R_{2}^{*}$ slightly, assessing the volume-averaged tissue iron concentration of dopaminergic neurons is in reach of *in vivo* MRI relaxometry. Strong contrast in $R_{2}^{*}$ was observed between the millimeter-thin N1 and the surrounding tissue with more than 40 % increase in $R_{2}^{*}$ in the DN-rich area. Hence, *in vivo* nigrosome characterization with 7T MRI requires quantitative maps of $R_{2}^{*}$ and $R_{2}$ with sub-millimeter resolution and signal-to-noise ratio (SNR) of at least 4 to achieve a contrast-to-noise ratio of 2. A multi-echo GE acquisition with a resolution of 500 micro metre resulting in $R_{2}^{*}$ maps with averaged SNR of about 20 was demonstrated at 7T *in vivo* (Tardif et al., 2016), opening the path for *in vivo* assessment of *substantia nigra*’s substructure.

## 5 Discussion

This work establishes a comprehensive biophysical model of iron-induced transverse and effective transverse relaxation rates in the nigrosomes of human *substantia nigra*. We have demonstrated that iron in neuromelanin-rich dopaminergic neurons is the predominant contrast driver in the nigrosomes (Fig. 1, Fig. 2). Using quantitative cellular iron maps and biophysical modeling, we predicted iron-induced relaxation rates from first principles and quantified the impact of different relaxation mechanisms induced by iron stored in two chemical forms (ferritin and neuromelanin). We characterized the distribution of iron in these two forms (Figs. 3, S6) and separately estimated their impact on quantitative MRI parameters. In nigrosome N1, most of the iron was bound to ferritin, with about 12 % stored in neuromelanin in DN (Table 2). Despite its lower total concentration, neuromelanin-bound iron was the major contributor to nigral $R_{2}^{*}$ relaxation, explaining 60 % of iron-induced relaxation rates in a representative volume of several MRI voxels within N1 (Fig. 5). Both quantitative biophysical modeling and qualitative assessment indicated that the heterogeneous cellular iron distribution on the microscale is the main effective transverse relaxation mechanism in N1. This contribution is well described by a static dephasing approximation (Fig. 4). The distribution of iron in DN on quantitative, MRI-derived concentration maps showed a high spatial correspondence to quantitative histological maps and provided an estimate of the iron concentration in the DN’s neuromelanin (Fig. 6).

### 5.1 Biophysical modeling informs the design of MRI-based biomarkers of nigrosome integrity

We propose two potential biomarkers of iron in DN: One based on the reversible portion of the iron-induced effective transverse relaxation rate ($R_{2}^{*}-R_{2}$) in N1 (Eq. (12)), the other on the difference in $R_{2}^{*}$ between N1 and the directly surrounding, DN-poor tissue (Eq. (12)). Both markers are driven by the volume-averaged tissue iron concentration of neuromelanin clusters, i.e. by the product of local iron concentration in DN and the density of DN (Yablonskiy and Haacke, 1994). We expect these relations to hold *in vivo*, as the predicted iron-induced relaxation rates decreased by merely 7 % due to temperature and tissue fixation effects (Fig. S8). These potential biomarkers of the volume-averaged tissue iron concentration in DN are likely informative because the density of DN and their iron load strongly varies across the SN and also between individuals (Figs. 6, S2, S6). The biomarkers are expected to be sensitive to age-related iron accumulation in DN (Zecca et al., 2004a) and to DN depletion (Damier et al., 1999b). Therefore, the two proposed biomarkers may also be sensitive to PD and accompanying dysfunctions (Tambasco et al., 2019).

We recently demonstrated that in symptomatic PD patients, iron load of dopaminergic neurons may be doubled as compared to controls, based on the analysis of intracellular iron concentration in *post mortem* tissue (Friedrich et al., 2021). On the other hand, these neurons in N1 deplete in PD. Note that the impacts of these two processes on the proposed biomarkers counteract each other, as the biomarkers reflect the product of DN iron concentration and DN density. Informing our biophysical model with these histological results in PD, we can estimate the expected changes in both proposed biomarkers between healthy controls and PD patients. The study reported that merely 20 % of DN survived in N1 of PD patients, while the intraneuronal iron concentration increased by 62 % (Friedrich et al., 2021). Hence, the NM contribution to the first biomarker based on $R_{2}^{*}-R_{2}$ is reduced in PD by 70 % (0.2·1.62=0.3), or by $12.3\;s^{-1}$. However, this reduction may be partly reversed by the increased contribution from ferritin-bound iron, which was reported to increase by about 60 % in PD patients, resulting in an increase of the $R_{2}^{*}-R_{2}$ values in the nigrosomes by approximately $7\;s^{-1}$. This suggests that the first biomarker based on $R_{2}^{*}-R_{2}$ would be overall reduced by about $5\;s^{-1}$ in PD patients. The second biomarker, which is the difference in $R_{2}^{*}$ between neuron-rich N1 and the directly surrounding area is insensitive to the contribution of ferritin. For this biomarker, we expect a higher sensitivity (a 70 % decrease in the number of DN corresponds to a reduction of approximately $20\;s^{-1}$) and higher specificity (no impact from an increase of ferritin-bound iron, if the variation is small between N1 and its direct surrounding tissue). These estimates should be cautiously interpreted, since they are based on histological data from late stage PD patients suffering from severe DN losses but also severe iron accumulation due to neurodegeneration. Since the histological data for early stage patients are currently not available, an *in vivo* MRI study on a cohort including PD patients is needed to test the utility of the proposed biomarkers for diagnostic purposes.

Our generative biophysical model has fundamental implications for the understanding of relaxation mechanisms in the human brain. It demonstrates that knowledge about the cellular iron distribution and iron’s chemical form are indispensable for interpreting GE and SE signal decays in iron-rich brain regions. Current models of iron-induced MRI parameters (Haacke et al., 2005; Langkammer et al., 2012; Stüber et al., 2014; Yao et al., 2009) often oversimplify the impact of tissue iron by using a single empirical proportionality constant between the average tissue iron concentration and the MRI parameter across brain areas. For N1 of sample 1, where we found an average iron concentration of $60\;\mu {g\;g}^{-1}$, one such model (Stüber et al., 2014) predicts an $R_{2}^{*}$ of $17.7\;s^{-1}$, explaining less than half of the iron-induced $R_{2}^{*}=\left(42\pm 11\right)\;s^{-1}$. This was estimated as the difference between $R_{2}^{*}$ measured in this area (Fig. 5) and the average $R_{2}^{*}$ rate in N1 after iron extraction (Fig. 2D, Table 1). Our model is able to explain this difference by taking iron’s heterogeneous cellular distribution and chemical form into account, predicting a total iron-induced $R_{2}^{*}$ of $\left(49.5\pm 0.3\right)\;s^{-1}$. This stresses the importance of precise and specific models, as developed and presented here.

Our model predicts that the MRI parameters $R_{2}^{*}$, $R_{2}$, and the bulk susceptibility measured with QSM are all affected differently by neuromelanin- and ferritin-bound iron pools. For instance, iron in DN contributes 60 % of iron-induced $R_{2}^{*}$, but merely 29 % of iron-induced bulk susceptibility. Therefore, combining the information from all three parameters may enable the separate quantification of both iron pools using the quantitative links established by our model.

Our approach can be extended to study other iron-rich structures in the human brain. Only a few studies have addressed the microscopic mechanisms of iron’s contribution to $R_{2}^{*}$ of brain structure (Troprés et al., 2001; Wen et al., 2018). The contributions to $R_{2}^{*}$ of iron-rich glial cells in healthy gray and white matter, such as oligodendrocytes, micro- and astroglia, as well as iron in myelin sheaths, have not yet been systematically explored. Iron is known to accumulate in amyloid plaques and neurofibrillary tangles in Alzheimer’s disease (Meadowcroft et al., 2015) and in multiple sclerosis lesions (Craelius et al., 1982). Thus, the more refined understanding of the mechanisms underlying iron-induced relaxation we presented here opens the door to more specific diagnostic biomarkers in these disorders.

### 5.2 Our results in the context of previous work

The iron concentrations obtained in our study agree well with previous reports. To our knowledge, only two studies have reported local iron concentrations in dopaminergic neurons. In a single DN in SN, the local iron concentration was estimated to be $230\;\mu g\;g^{-1}$ (Morawski et al., 2005), while in a more recent study we reported a range of local iron concentrations in DN in nigrosome N1 from $85\;\mu g\;g^{-1}$ to $1371\;\mu g\;g^{-1}$ (values are corrected for volume shrinkage as in the present study)^5^ Friedrich et al. (2021). Both prior results agree with the range of local iron concentrations in DN from the present study (Figs. 3A, S6). The sum of volume-averaged tissue iron concentrations in neuromelanin and ferritin in SN (in sample 1, 2, and 3 $\left(63.0\pm 2.5\right)\;\mu g\;g^{-1}$, $\left(201.1\pm 1.2\right)\;\mu g\;g^{-1}$, and $\left(573\pm 4\right)\;\mu g\;g^{-1}$, respectively) is on the order of the reported iron concentrations averaged across the entire SN, $48\;\mu g\;g^{-1}\;to\;204\;\mu g\;g^{-1}$ (Dexter, Carayon, Javoy-Agid, Agid, Wells, Daniel, Lees, Jenner, Marsden, 1991, Dexter, Wells, Lee, Agid, Agid, Jenner, Marsden, 1989; Friedrich et al., 2021; Galazka-Friedman et al., 1996; Hallgren and Sourander, 1958; Loeffler et al., 2002; Morawski et al., 2005; Riederer et al., 1989; Zecca et al., 2004a).

Increased $R_{2}^{*}$ relaxation rates in the nigrosomes are in line with recent studies (Lee, Baek, Chun, Lee, Cho, 2018, Lee, Baek, Song, Lim, Lee, Nguyen, Kim, Huh, Chun, Cho, 2016; Lee, Baek, Chun, Lee, Cho, 2018, Lee, Baek, Song, Lim, Lee, Nguyen, Kim, Huh, Chun, Cho, 2016). An $R_{2}^{*}$ of $\left(82\pm 25\right)\;s^{-1}$ was observed in the nigrosomes in the first sample of our study (Fig. 2D), which corresponds well to a reported $R_{2}^{*}$ of $\left(103\pm 3\right)\;s^{-1}$ in neuromelanin-rich regions within SN in *post mortem* tissue (Arai et al., 2020; Jin et al., 2019). In all examined samples, the neuromelanin-rich nigrosomes showed increased $R_{2}^{*}$, after registering $R_{2}^{*}$ maps to histology using ultra-high resolution $T_{2}^{*}$-WI (Figs. 1A, B, C; S2A, B, D). The $R_{2}^{*}$ relaxation rates in samples 2 and 3 were higher than in sample 1, which can be attributed to intersubject variability of DN neuromelanin local iron concentration and volume fraction (Table 2, Fig. S6). In all investigated samples, we observed that the histologically defined nigrosome 1 corresponded to a thin stripe with increased $R_{2}^{*}$ compared to surrounding tissue (Figs. 1A, B, C; S2A, B, D). This robust experimental observation challenges the widespread interpretation of *in vivo* MRI data, where a much larger area showing decreased $R_{2}^{*}$ compared to its surrounding is commonly identified with nigrosome 1 (Kim et al., 2019; Lehéricy et al., 2014; Mahlknecht et al., 2017; Schwarz, Afzal, Morgan, Bajaj, Gowland, Auer, 2014, Schwarz, Mougin, Xing, Blazejewska, Bajaj, Auer, Gowland, 2018). Based on a *post mortem* study by Blazejewska et al. (2013), which used the established histological definition of the nigrosomes and co-registration between histology and MRI, recent MRI literature substituted the histological definition of nigrosome 1 with an MRI-based definition, using nigrosome 1 as a synonym for the hypointense part of the swallow tail sign or the dorsal nigral hyperintensity (Arai et al., 2020; Jin et al., 2019; Kim et al., 2019). Our results consistently showed that at least in the posterior dorsal part of *post mortem* SN, the wide-spread MRI-based definition of N1 is inaccurate. A reason for the differing interpretation and segmentation may be a difference in co-registration strategies between our study and Blazejewska et al. (2013). Our local co-registration between MRI and histology achieved a high precision of 100 micro meter, relying on small vessels around SN as landmarks and a similarity transformation. The earlier study used an affine co-registration of large tissue sections and lower resolution MRI data, allowing for shear to compensate for tissue deformation during sectioning, which potentially caused a local mismatch. The posterior-dorsal part of nigrosome 1 has a thickness of about 1 mm and differs in its extent and geometry from the swallow tail sign. Further studies combining MRI and histology in 3D would help to elucidate the exact relation between histologically defined nigrosome 1 and the swallow tail sign by comparing histologically defined nigrosome atlases to MRI contrast (Alkemade et al., 2020; Brammerloh et al., 2021).

It was recently reported that the swallow tail sign shows intersubject variability in *in vivo* MRI data (Cheng et al., 2019). Indeed, SN contains a complex arrangement of sub-millimeter-sized structures within a volume of less than a cubic centimeter: iron-rich and iron-poor myelinated fibers, GABAergic and dopaminergic neuron clusters, and an anterior-posterior iron accumulation gradient (Massey et al., 2017). The contributions of all these tiny structures to quantitative MRI parameters including $R_{2}^{*}$, $R_{2}$, longitudinal relaxation rate ($R_{1}$) and magnetization transfer (MT) are still unclear. The interplay of these physical parameters needs to be understood to improve the interpretation of SN’s appearance in MR images with different weightings at clinically achievable resolution. For example, Massey et al. (2017) recently demonstrated that some nigrosomes appear hyperintense on turbo-spin-echo images, combining $R_{1}$, $R_{2}$, and MT weighting, even after neuromelanin-rich neurons were largely depleted in the brain of PD patients. Unfortunately, this study did not include a $R_{2}^{*}$ mapping, such that the biomarkers we proposed and the correspondence of swallow tail and nigrosomes in 3D are difficult to compare.

A further study is required to identify the histological underpinning of the swallow tail sign and its exact relation to N1, including precisely co-registered multi-modal quantitative MRI and histology on whole brains. Such a study would be of high importance for the development of an *in vivo* nigral biomarker, since the *substantia nigra* is a heterogeneous structure, containing not only the nigrosomes but also afferent and efferent fibers. As relaxation is impacted by different structures across SN, it will be crucial to find the regions where dopaminergic neurons contribute predominantly.

### 5.3 Experimental limitations and biophysical modeling assumptions

The biophysical model developed in this study was derived from first principles, but relies on several assumptions about the cellular distribution of iron and on literature-derived magnetic and MR-properties of ferritin- and neuromelanin-bound iron obtained from *in situ* experiments. Moreover, the model was informed and validated by histology and MRI on *post mortem* tissue, which may differ from *in vivo* in several ways. The resulting experimental and theoretical limitations in our approach are discussed in the following.

The minor effects of increased temperature and diffusion coefficient (Birkl et al., 2016) *in vivo* were already discussed.

Our model probably underestimates iron-induced relaxation *in vivo* by 5 %, as the labile iron pool is washed out during preparation, before PIXE measurements and histochemistry are performed (Kakhlon and Cabantchik, 2002; Stüber et al., 2014).

In the chemical iron extraction experiment, which we used to quantify the iron-induced relaxation in SN, we assumed that all changes in MRI parameters are attributed to missing iron. Alterations of non-iron-induced relaxation rates most likely did not affect $R_{2}^{*}$, as we found no significant differences between $R_{2}^{*}$ pre- and post-extraction in the iron-poor crus cerebri region on a quantitative $R_{2}^{*}$ map (Fig. 2B, ventro-lateral of ROI S).

We modeled the nanoscale relaxation using relaxivities estimated from *in vitro* measurements (Gossuin et al., 2002; Trujillo et al., 2017), which may systematically differ from relaxivities in tissue. For example, $R_{2}$ in ferritin solutions depends on temperature (changing by about 10 % between room and body temperature Gossuin et al. (2000)) and pH (about 3 % variation within physiological pH Gossuin et al. (2000)). Particularly for neuromelanin, the difference in molecular structure and granularity of the synthetic melanin used *in vitro* may affect its effective relaxivity. Additionally, the assumed linear dependence of $R_{2}$ relaxivity on the magnetic field, known from other iron-containing particles, may not hold for neuromelanin, potentially introducing a bias in our estimations. An accurate *in vivo* measurement or gold standard model system of neuromelanin related relaxivity has not been achieved yet. While the iron extraction experiment showed that iron contributes much stronger to $R_{2}^{*}$ than to $R_{2}$, nanoscale processes contribute equally to $R_{2}$ and $R_{2}^{*}$. Therefore, a change in nanoscale relaxation rates does not alter our main conclusions regarding the relaxation mechanisms in N1.

To model relaxation rates due to microscale processes, we estimated the effective susceptibility per iron load of DN using Curie’s law for an isolated spin 5/2, which is an oversimplification in view of the two iron binding sites of neuromelanin (Zucca et al., 2017). An experiment to determine neuromelanin’s susceptibility would help us to refine our model even further.

While the high correspondence between experimental results and theory makes it unlikely that any major contributor was overlooked, relaxation effects due to more fine-grained iron distribution patterns on the intermediate length scale of several hundred nanometers, smaller than the voxel size of the 3D iron concentration map, were not modeled. The 3D iron concentration maps had an in-plane resolution of 0.88 micro meter and a slice thickness of 10 micro meter, which could be increased using electron microscopy. Nevertheless, processes on the intermediate length scale would likely contribute equally to $R_{2}^{*}$ and $R_{2}$, and are hence not a suitable candidate to explain the iron-induced $R_{2}^{*}$ in N1.

The model did not explicitly include myelin as a driver of $R_{2}^{*}$ and $R_{2}$ contrast, since the myelin concentration in N1 is low, as can be seen on Luxol stains for myelin (Fig. S2F1). Using the model in other brain areas will require taking myelin’s contribution into account.

In this study, we determined that a biomarker of iron in DN is within reach of state-of-the-art MRI methods (Tardif et al., 2016). Recently developed methods for prospective motion correction and physiological noise correction (Stucht et al., 2015; Vannesjo et al., 2015; Versluis et al., 2010) promise to improve data quality even further (Metere et al., 2017; Trampel et al., 2019). Yet, we compared theoretical predictions to experimental values in a region spanning only four MRI voxels. The region was limited by the area of neuron-to-neuron registration, and comprised a volume of 440μm×440μm×100μm. Therefore, the relative contributions of different relaxation mechanisms, reported in Fig. 5, correspond to few representative voxels and were not averaged across nigrosomes. To extend the theory to other regions in SN, the comparison may be performed on a larger region. This would require the challenging co-registration of the entire SN by identifying shared DN on sections stained with Perls’ solution for iron.

## 6 Conclusion

In this paper, we have introduced a generative model of iron-induced transverse relaxation in nigrosome 1, informed by 3D quantitative iron histology. Our biophysical model constitutes an important step on the road toward a unified, quantitative understanding of iron-induced MRI relaxation in the human brain. We demonstrate that and explain why dopaminergic neurons contribute predominantly to iron-induced $R_{2}^{*}$ in N1, although their neuromelanin contains only a minority of tissue iron. By linking $R_{2}^{*}$ to the tissue iron concentration in dopaminergic neurons, this study lays the groundwork for developing a biomarker of nigral integrity. Such a biomarker would help in understanding the relationship between iron accumulation and neuronal depletion in healthy aging and Parkinson’s disease.

## Data and code availability statement

All data required to support the conclusions in the paper are given in the paper and/or the Supplementary Materials. Additional data related to this paper may be obtained from the authors upon request. Only fully anonymized data can be provided. The code used in this study is publicly available on https://www.cbs.mpg.de/departments/neurophysics/software/genr2s.

## Declaration of competing interest

The Max Planck Institute for Human Cognitive and Brain Sciences has an institutional research agreement with Siemens Healthcare. NW was a speaker at an event organized by Siemens Healthcare and was reimbursed for the travel expenses. NW holds a patent on acquisition of MRI data during spoiler gradients (US 10,401,453 B2).

## CRediT authorship contribution statement

**Malte Brammerloh:** Methodology, Software, Formal analysis, Investigation, Data curation, Writing - original draft, Visualization, Project administration, Funding acquisition. **Markus Morawski:** Conceptualization, Methodology, Investigation, Validation, Resources, Writing - review & editing, Funding acquisition. **Isabel Friedrich:** Methodology, Investigation, Writing - review & editing. **Tilo Reinert:** Methodology, Investigation, Data curation, Writing - review & editing. **Charlotte Lange:** Investigation, Data curation, Writing - review & editing. **Primož Pelicon:** Resources, Methodology, Investigation, Writing - review & editing, Funding acquisition. **Primož Vavpetič:** Resources, Methodology, Investigation, Writing - review & editing, Funding acquisition. **Steffen Jankuhn:** Methodology, Investigation, Data curation, Writing - review & editing. **Carsten Jäger:** Methodology, Investigation, Validation, Writing - review & editing. **Anneke Alkemade:** Methodology, Investigation, Resources, Writing - review & editing. **Rawien Balesar:** Methodology, Investigation, Writing - review & editing. **Kerrin Pine:** Methodology, Data curation, Software, Writing - review & editing. **Filippos Gavriilidis:** Investigation, Writing - review & editing. **Robert Trampel:** Methodology, Software, Writing - review & editing. **Enrico Reimer:** Software, Writing - review & editing. **Thomas Arendt:** Resources, Writing - review & editing, Funding acquisition. **Nikolaus Weiskopf:** Conceptualization, Resources, Methodology, Writing - review & editing, Supervision, Project administration, Funding acquisition. **Evgeniya Kirilina:** Conceptualization, Methodology, Software, Formal analysis, Investigation, Data curation, Writing - original draft, Visualization, Supervision, Project administration.

## Appendix A Supplementary materials

Supplementary Data S1 Supplementary Raw Research Data. This is open data under the CC BY license http://creativecommons.org/licenses/by/4.0/ Supplementary Data S1

Supplementary Data S2 Supplementary Raw Research Data. This is open data under the CC BY license http://creativecommons.org/licenses/by/4.0/ Supplementary Data S2

Supplementary Data S3 Supplementary Raw Research Data. This is open data under the CC BY license http://creativecommons.org/licenses/by/4.0/ Supplementary Data S3

Supplementary Data S4 Supplementary Raw Research Data. This is open data under the CC BY license http://creativecommons.org/licenses/by/4.0/ Supplementary Data S4

## Footnotes

2 The nanoscale relaxivity of ferritin was estimated using relaxation rates reported in Gossuin et al. (2000) for ferritin in solution with iron concentration of $1^{-1}$ at 40${}^{\circ }$C. By evaluating the linear relation between $R_{2}$ and Larmor frequency f presented in Fig. 1A in Gossuin et al. (2000), we obtained $R_{2}=5.27\;s^{-1}+0.39\;s^{-1}/\mathrm{MHz}\cdot 298\;\mathrm{MHz}=120\;s^{-1}$, for Larmor frequency f=298 MHz corresponding to 7T MRI. The obtained relaxation rate was converted to a relaxivity by dividing it by the used iron concentration ($100\;\mathrm{mmol}1^{-1}$) converted from mmol$1^{-1}$ to μg g${}^{-1}$ using the molar weight of iron of $56\;{g\;\text{mol}}^{-1}$ and a water density of 1 kg$1^{-1}$. This results in $r_{2,\mathrm{FT}}=0.02\;s^{-1}/\mu g/g$.

3 We estimated the $r_{2,\mathrm{NM}}$ at 7T using relaxivity values of $0.363\;s^{-1}\mu g/g$ obtained at 3T for iron-loaded synthetic melanin immobilized in gels (Trujillo et al., 2017) and suggesting the same linear dependence of the relaxivity on the magnetic field as for iron oxide particles (Gossuin et al., 2002). Scaling the relaxivity values linearly from 3T to 7T, we obtained the value $r_{2,\mathrm{NM}}=0.8\;s^{-1}\mu g/g$.

4 This was estimated using Curie’s law: $\chi_{in\;vivo}/\chi_{post\;mortem}=T_{post\;mortem}/T_{in\;vivo}=293\;K/310\;K\approx 95\;\%$.

5 To ensure comparability, we applied the same tissue shrinkage correction as for our PIXE measurements to the reported value, which was a factor of 0.76.
