# Supplementary material for: Measuring the iron content of dopaminergic neurons in substantia nigra with MRI relaxometry
Source: Neuroimage. 2021 Oct 1;239:118255. doi: 10.1016/j.neuroimage.2021.118255 (PMC8363938; doi:10.1016/j.neuroimage.2021.118255)
Supplement: Supplementary Data S1 — Supplementary Raw Research Data. This is open data under the CC BY license http://creativecommons.org/licenses/by/4.0/ [file mmc1.pdf]

Measuring the iron content of dopaminergic neurons in substantia nigra with MRI relaxometry
Supplementary Information

Malte Brammerloh,[1,2,3,*] Markus Morawski,[4] Isabel Friedrich,[4] Tilo Reinert,[1,3] Charlotte Lange,[1,3] Primož Pelicon,[5] Primož Vavpetič,[5] Steffen Jankuhn,[3] Carsten Jäger,[1] Anneke Alkemade,[6] Rawien Balesar,[6] Kerrin Pine,[1] Filippos Gavriilidis,[1] Robert Trampel,[1] Enrico Reimer,[1] Thomas Arendt,[4] Nikolaus Weiskopf,[1,3,†] and Evgeniya Kirilina[1,7,†]

[1]*Department of Neurophysics, Max Planck Institute for Human Cognitive and Brain Sciences, Stephanstr. 1a, 04103 Leipzig, Germany*

[2]*International Max Planck Research School on Neuroscience of Communication: Function, Structure, and Plasticity*

[3]*Felix Bloch Institute for Solid State Physics, Faculty of Physics and Earth Sciences, Leipzig University, Linnéstr. 5, 04103 Leipzig, Germany[*]*

[4]*Paul Flechsig Institute of Brain Research, University of Leipzig, Liebigstr. 19, 04103, Leipzig, Germany*

[5]*Jožef Stefan Institute, Jamova 39, SI-1000 Ljubljana, Slovenia*

[6]*Integrative Model-based Cognitive Neuroscience Research Unit, University of Amsterdam, Amsterdam, Nieuwe Achtergracht 129B, 1001 NK Amsterdam, The Netherlands*

[7]*Center for Cognitive Neuroscience Berlin, Free University Berlin, Habelschwerdter Allee 45, 14195, Berlin, Germany*

ESTIMATION OF THE SUSCEPTIBILITY OF NEUROMELANIN-BOUND IRON

A monoatomic iron binding site with spin of 5/2 was observed within neuromelanin in *substantia nigra* using electron paramagnetic resonance spectroscopy [1]. We therefore estimated neuromelanin's susceptibility per iron load by using Curie's law for monoatomic iron with spin $S = 5/2$.

$$\chi_{\mathrm{NM}} = \frac{\mu_0 S(S+1) g^2 \mu_\mathrm{B}^2}{3 k_\mathrm{B} T} = 3.275\,\mathrm{ppbg/\mu g},$$

where μ_0 is the vacuum permeability, $g = 2$ the Landé factor of the electron, μ_B the Bohr's magneton, k_B Boltzmann's constant and $T = 300\,\mathrm{K}$ the temperature. Note, that this value provides only a coarse approximation of the magnetic susceptibility of neuromelanin-bound iron since the structure of NM is known to be complex. Crystalline domains with unknown susceptibility were reported to be present within NM granules [2].

[1] L. Zecca, A. Stroppolo, A. Gatti, D. Tampellini, M. Toscani, M. Gallorini, G. Giaveri, P. Arosio, P. Santambrogio, R. G. Fariello, E. Karatekin, M. H. Kleinman, N. Turro, O. Hornykiewicz, and F. A. Zucca, The role of iron and copper molecules in the neuronal vulnerability of locus coeruleus and substantia nigra during aging, Proceedings of the National Academy of Sciences of the United States of America **101**, 9843 (2004).

[2] D. Sulzer, C. Cassidy, G. Horga, U. J. Kang, S. Fahn, L. Casella, G. Pezzoli, J. Langley, X. P. Hu, F. A. Zucca, I. U. Isaias, and L. Zecca, Neuromelanin detection by magnetic resonance imaging (MRI) and its promise as a biomarker for Parkinson's disease, NPJ Parkinson's Disease **4**, 10.1038/s41531-018-0047-3 (2018).

[3] P. Damier, E. C. Hirsch, Y. Agid, and A. M. Graybiel, The substantia nigra of the human brain. I. Nigrosomes and the nigral matrix, a compartmental organization based on calbindin D(28K) immunohistochemistry., Brain **122**, 1421 (1999).

[4] B. B. Avants, N. J. Tustison, G. Song, P. A. Cook, A. Klein, and J. C. Gee, A Reproducible Evaluation of ANTs Similarity Metric Performance in Brain Image Registration, NeuroImage **54**, 2033 (2011).

* mbrammerloh@cbs.mpg.de

† N.W. and E.K. contributed equally to this work.

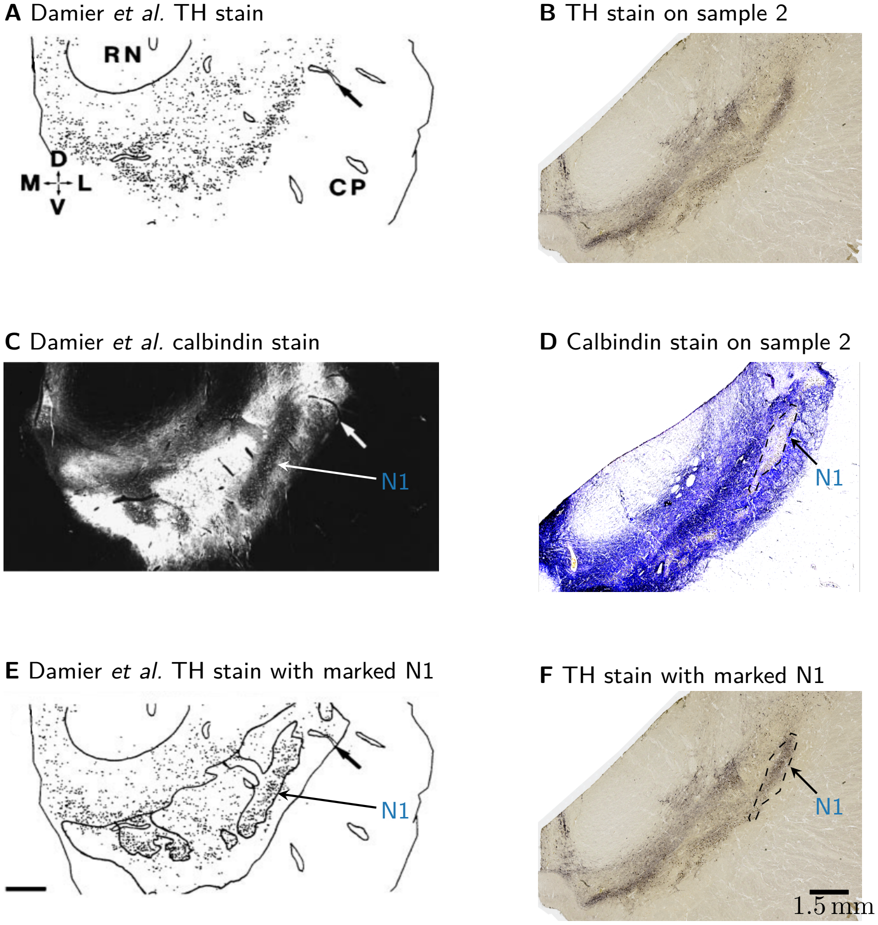

Figure S1. Nigrosome 1 (N1) definition following Damier *et al.* [3]. A: On sections stained for tyrosine hydroxylase (TH), Damier *et al.* marked the locations of the somata of dopaminergic neurons with black dots (A, C, and D adapted from [3]). B: A section from sample 2 of our study stained for TH shows a stripe of high staining intensity with similar geometry as in A. C: On sections stained for calbindin, Damier *et al.* identified nigrosomes as areas of low staining intensity. N1, the largest nigrosome, was defined as a large structure extending along the medioventral-dorsolateral axis. D: A section stained for calbindin from sample 2 shows an elongated structure of low staining intensity as C, which we identified as N1. The N1 area is marked with a dashed line. E: The nigrosome delineations of low calbindin immunoreaction (from C) are overlayed on the TH-positive DN (from A). Inside the nigrosomes, Damier *et al.* found an increased density of DN [3]. F: The calbindin-poor N1 region (from D) is overlaid on the section stained for TH (B), showing high TH immunoreaction in N1 as in E. On A, anatomical directions are indicated as medial (M), lateral (L), ventral (V), and dorsal (D).

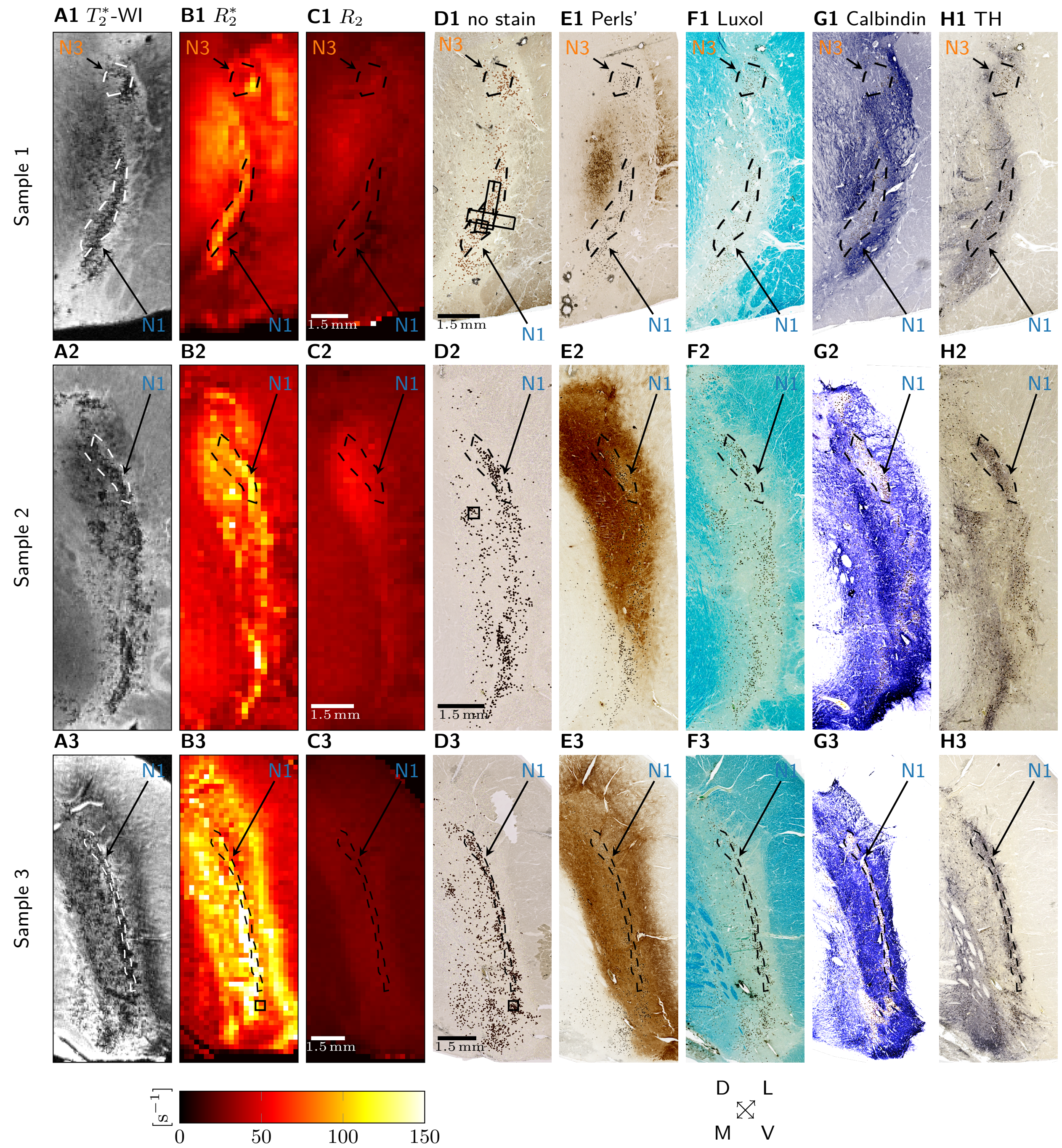

Figure S2. (Caption next page.)

Figure S2. MRI and histology data on the three analyzed samples are presented in three rows. A1-3: T_2^*-WI show granular hypointensities in the nigrosomes. B1-3: On quantitative R_2^* maps, areas with high relaxation rates resemble the hypointensities in T_2^*-WI. Videos of the R_2^* maps, available in the online version of this article, show the 3D structure of this area. C1-3: On quantitative R_2 maps, no nigrosome structure is visible. An area of slightly increased R_2 resembles the shape of high Perls' staining intenisty (E1-3). D1-3: Clusters of high neuromelanin density on unstained sections were co-localized with granular hypointensities in T_2^*-WI and hyperintensities on quantitative R_2^* maps. (Each neuromelanin domain was marked with a brown dot for better visibility.) The PIXE measurement areas (Figs. 1, 3, S5) are indicated with black squares. In sample 1, the dashed and solid squares indicate measurements on two adjacent histological sections. E1, E2, E3: On sections stained with Perls' solution for iron, a high intersubject variability was observed. F1-3: On sections stained with Luxol for myelin, a low staining intensity was observed in the SN, including the nigrosome areas. G1-3: On sections stained for calbindin, an elongated structure of low staining intensity was identified as N1 and marked with a dashed line. For sample 1, a circular structure of low staining intensity was identified as N3 and marked with a dashed line as well. H1, H2, H3: On sections stained for tyrosine hydroxylase (TH), the regions of high staining intensity showed high spatial correspondence to the distribution on neuromelanin on the unstained sections (D1-3). In all images, the location of N1 is indicated with an arrow and a dashed line encompasses the N1 area identified on the sections stained for calbindin. Anatomical directions are indicated as medial (M), lateral (L), ventral (V), and dorsal (D).

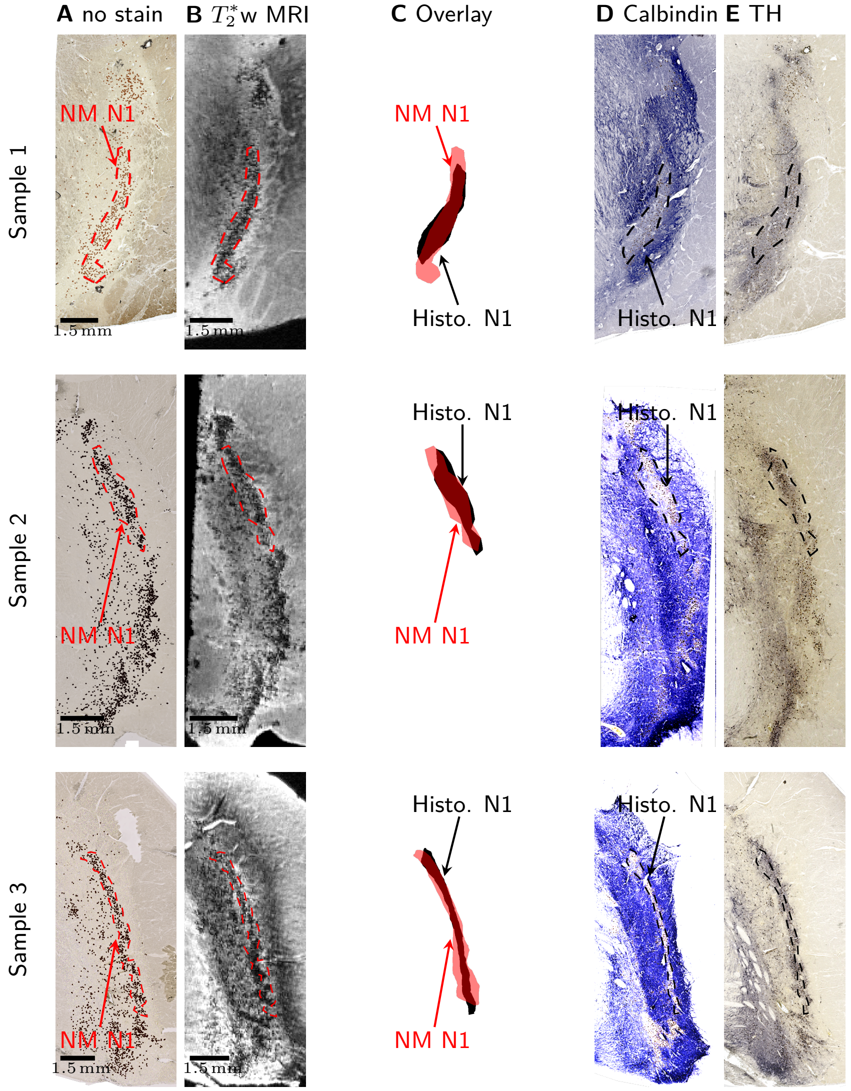

Figure S3. Neuromelanin-based N1 and histochemical N1 show high overlap in *post mortem* SN. A: A stripe with high density of iron-rich neuromelanin (NM) domains was used to define NM-based N1. B: At the location of the NM-based N1, T_2^*-WI shows a stripe of granular hypointensities, caused by the high content of paramagnetic iron in NM. C: Overlaying the NM-based N1 with the histochemical N1, the NM-based N1 covered $(84 \pm 2)\,\%$ of the histochemically defined N1. The intersection of the NM-based and histochemical N1 covered $(66 \pm 5)\,\%$ of the NM-based N1's area. Based on this high correspondence between the histochemical and NM-based N1 areas, we identified N1 as a stripe of granular hypointensities on high-resolution T_2^*-WI on the sample that was used in the tissue iron extraction experiment. D: The histochemical N1 was delineated as an elongated stripe of low calbindin immunoreaction in the dorso-lateral SN with the geometry described by Damier *et al.* [3]. E: A strong TH immunoreaction was seen in the histochemical N1.

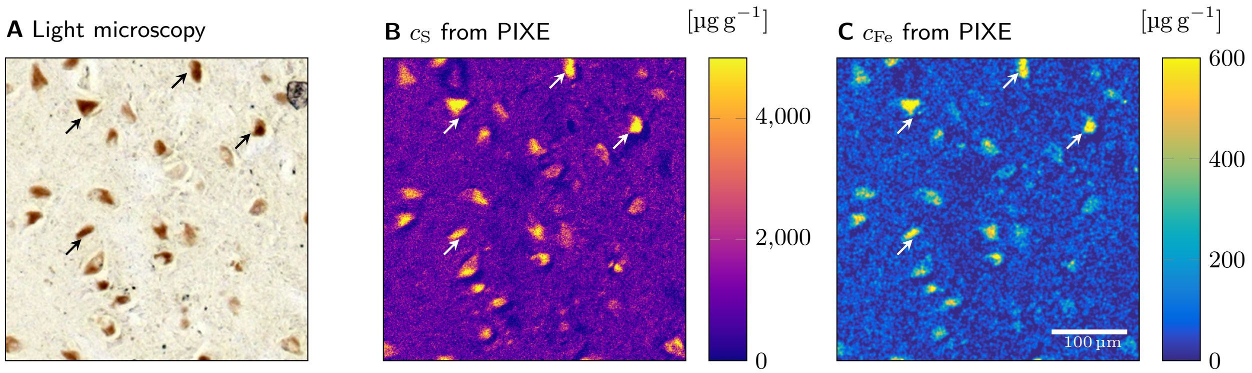

Figure S4. Co-registration of histological sections with PIXE data. A: Neuromelanin pigment is seen on unstained tissue sections in light microscopy. B: On quantitative sulfur concentration maps acquired with PIXE, the same neuromelanin clusters are seen as areas of increased concentration. An affine registration was performed to register the optical microscopy to the sulfur map using ANTs [4]. C: On quantitative iron concentration maps acquired with PIXE, the areas of elevated iron concentration show a high correspondence to the areas of elevated sulfur concentration, i.e. the location of neuromelanin domains in dopaminergic neurons. Four neuromelanin domains in dopaminergic neurons with a strongly increased iron concentration are marked with arrows.

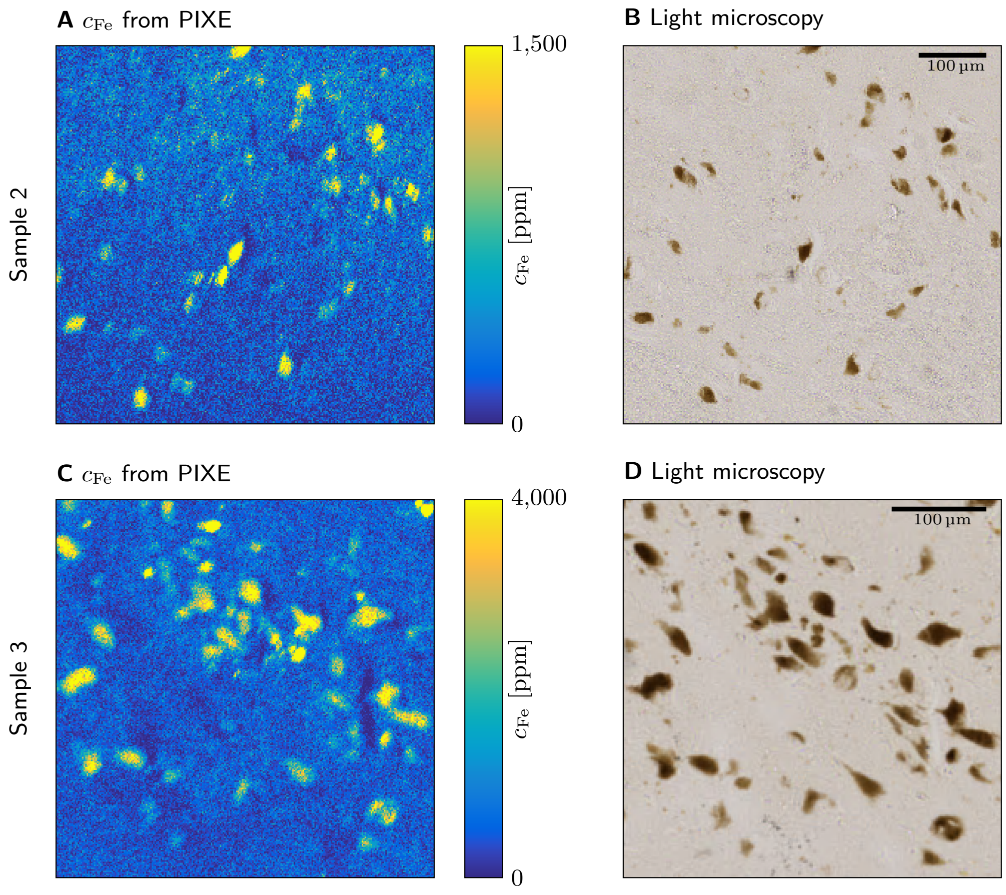

Figure S5. Iron concentration maps obtained with PIXE (A, C) together with light microscopy of the measurement area (B, D) in samples 2 (top row) and 3 (bottom). In all samples, neuromelanin domains in DN showed increased iron concentrations, while most iron was found outside of DN, probably associated with ferritin. In Table II, we report the average iron concentration in neuromelanin-rich areas of DN as well as in the rest of the measurement area, the volume fraction of neuromelanin-rich areas, and the iron fraction in DN. The depicted areas are indicated in Fig. S2D2, D3.

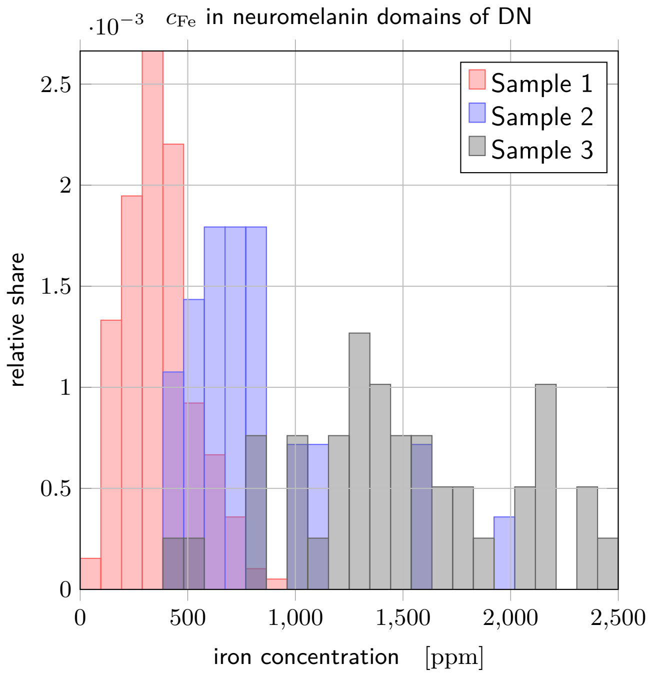

Figure S6. Histograms of iron concentrations in the neuromelanin of DN for all three samples. A high intersubject variability of $c_{\mathrm{Fe,NM}}$ in DN is apparent: For sample 1, a mean and standard deviation of (365 ± 161) ppm was found, for sample 2 (811 ± 366) ppm, for sample 3 (1495 ± 499) ppm, where the mean and standard deviation are calculated across neurons. These mean values are different from the values reported in Table II, because here each DN was weighted equally, while in the mean values in Table II the iron concentration is weighted with the DN's area in the microscopy section.

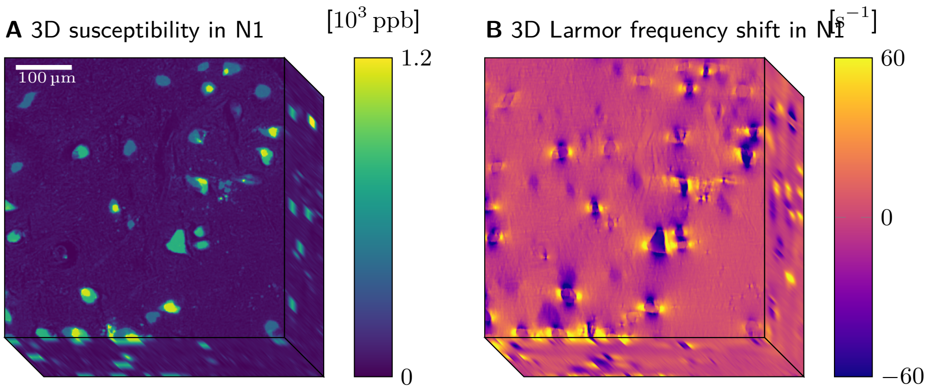

Figure S7. 3D susceptibility (A) and Larmor frequency shift maps (B) generated from the 3D quantitative iron map (Fig. 3D). DN show increased susceptibility and induce strong Larmor frequency perturbations in their vicinity.

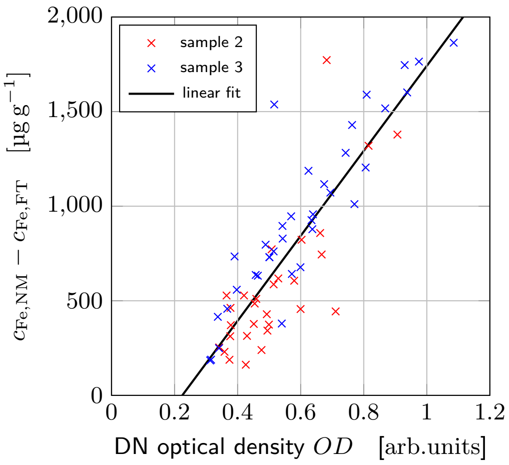

Figure S8. Optical density predicts iron concentration in DN measured with PIXE. The optical density in DN in the PIXE measurement areas on unstained sections from samples 2 and 3 was obtained from Eq. (10) with a $GS_{\mathrm{bg}} = 195$. The OD data points represent optical densities averaged over the area of individual DN in the PIXE measurement area. The iron concentration in these DN was obtained by averaging the PIXE iron concentration maps over the area of individual DN. Subsequently, the average iron concentration outside of the DN in the PIXE measurement areas of samples 2 and 3 ($c_{\mathrm{Fe,FT}}$, Table II) were subtracted from these iron concentrations in DN from samples 2 and 3, respectively. Correlating optical densities and iron concentrations in DN of samples 2 and 3, a Pearson correlation coefficient of $r = 0.85$ was found, with an associated $p = 8.32 \times 10^{-20}$. Fitting the linear relation $c_{\mathrm{Fe,NM}} = A \cdot OD + B$ to the data of both samples resulted in $A = (2250 \pm 180)\,\mu\mathrm{g\,g}^{-1}\,\mathrm{arb.units}^{-1}$ and $B = (-500 \pm 110)\,\mu\mathrm{g\,g}^{-1}$. This relation was used to generate maps of the volume-averaged iron concentration in the neuromelanin of DN on whole histological sections of samples 2 and 3 in Section 4.5 of the manuscript.

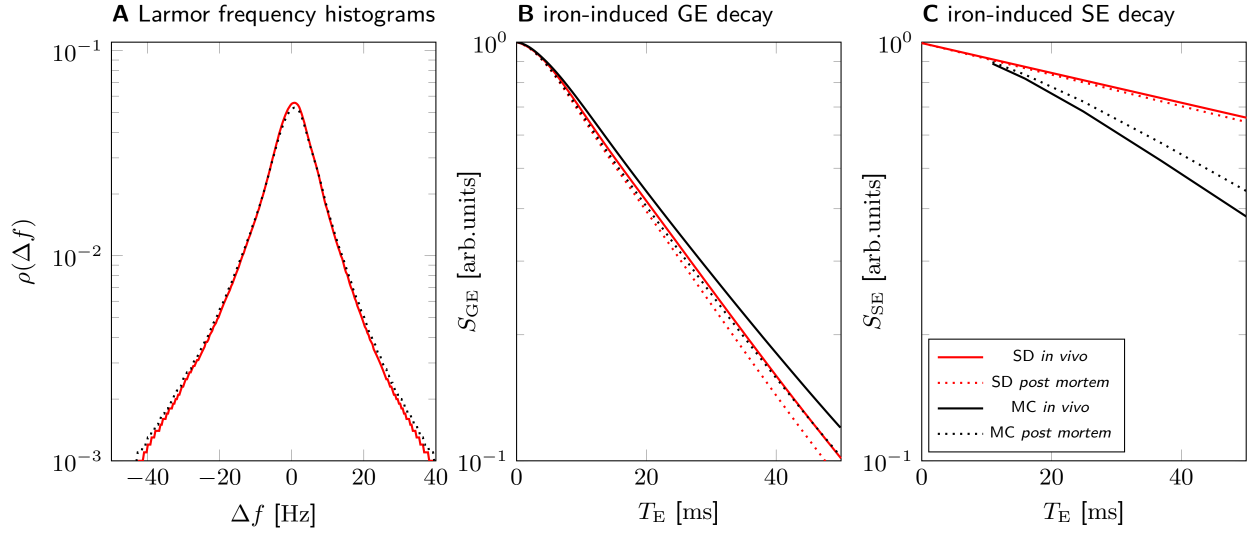

Figure S9. Modeling microscale relaxation in N1 of sample 1 in *in vivo* conditions. A: The increased temperature *in vivo* (310 K) reduces the width of the Larmor frequency shift histogram (red) slightly when compared to the *post mortem* histogram (black, dotted) (by $1 - 293\,\mathrm{K}/310\,\mathrm{K} \approx 5\,\%$). The susceptibility causes this because it is inversely proportional to the temperature according to Curie's law. B: Despite the higher temperature and faster diffusion *in vivo* (solid lines), the GE decays predicted with Monte Carlo (MC, black) and in static dephasing (SD, red) are similar to the ones predicted for *post mortem* conditions (dotted). Hence, the relaxation regime is also *in vivo* close to static dephasing. C: The SE decay predicted by MC in the *in vivo* condition is faster (black, solid) than *post mortem* (black, dotted).
